# Strong Photocurrent Response of Selenoarsenates With Different Transition Metal Complexes as Structure-Directing Agents

**DOI:** 10.3389/fchem.2022.890496

**Published:** 2022-05-05

**Authors:** Xinyu Tian, Gele Teri, Muge Shele, Namila E, Liming Qi, Min Liu, Menghe Baiyin

**Affiliations:** Inner Mongolia University Key Laboratory of Advanced Materials Chemistry and Devices (AMC&DLab), College of Chemistry & Environmental Science, Inner Mongolia Normal University, Hohhot, China

**Keywords:** solvothermal method, selenoarsenates, crystal structure, photocurrent responses, transition metal complex

## Abstract

Four selenoarsenates with different transition-metal complexes [Co(tren)_2_H]AsSe_4_ [tren = tris(2-aminoethyl)amine] (**1**); [Ni_2_(dien)_4_](As_2_Se_5_) (dien = diethylenetriamine) (**2**); [Zn(tren)]_2_(As_2_Se_5_) (**3**) and [Mn(tren)]_2_(As_2_Se_5_) (**4**) were solvothermally synthesized in a mixed solvent of organic amine and alcohol solution. The compounds **1-4** have pyramidal/tetrahedral structures (AsSe_3_/AsSe_4_), and contain transition metal (Co^2+^, Ni^2+^, Zn^2+^ and Mn^2+^) complex that form distinct zero-dimensional (0-D) clusters. Arsenic atoms form a tetrahedron in compounds **1** and **2**; **1** consists of discrete tetrahedral (AsSe_4_) and transition metal complex [Co(tren)_2_]^2+^; **2** is composed of an anion [As_2_Se_5_]^4-^ cluster and transition metal complex [Ni(dien)_2_]^2+^. In compounds **3** and **4**, arsenic atom forms a pyramidal AsSe_3_ and the two pyramidal AsSe_3_ share a corner connection to form a dimer [As_2_Se_5_]^4-^; **3** is characterized as a cluster consisting of two unsaturated [Zn(tren)]^2+^ caiton linked by a dimer (As_2_Se_5_)^4-^ linkage; in **4**, unsaturated [Mn(tren)]^2+^ caiton is linked to two trigonal-bipyramidal [Mn(tren)]Se via dimer (As_2_Se_5_)^4-^ to form [Mn(tren)]_4_[As_4_Se_10_] cluster. To our knowledge, [Zn(tren)]_2_(As_2_Se_5_) (**3**) is the first zinc selenoarsenate containing the (As_2_Se_5_)^4-^ anion type. Furthermore, the Mn^2+^ ions adopt a trigonal-biyramidal (five-coordinate) and octahedral (six-coordinate) environment. Adding K_2_CO_3_/Cs_2_CO_3_ to the synthesis system is necessary and may act as a mineralizer. Several properties of compounds **1**-**4** have been characterized in our studies, in particular their strong photocurrent response characteristics under visible light irradiation.

## Introduction

The wide variety of structures and properties of chalcogenidoarsenates have led to great interest in many areas, including semiconductors, photoelectricity, magnetism, ion exchange, and nonlinear optics (Sheldrick and Wachhold, 1998; Zhou et al., 2009; Zhang et al., 2012; Xiong et al., 2013; Yao et al., 2013; Liu et al., 2014; Zhou et al., 2015; Zhou, 2016; An et al., 2017; Wang et al., 2020; Chen et al., 2021; Li et al., 2021; Liu et al., 2021). The chalcogenidoarsenates are formed by corner- or edge-sharing of [AsQ_3_]^3-^ and [AsQ_4_]^3-^ (Q = S, Se) units, resulting in a variety of chalcogenidoarsenate aggregates. Such as [As_2_Q_4_]^2-^ (Smith et al., 1996; Xu et al., 2021), [As_2_Se_5_]^4-^ (Fu et al., 2006; Jia et al., 2006; Chen et al., 2021), [As_2_Se_6_]^2-^ (Wendel and Müller, 1995; Smith et al., 1998; Fu et al., 2005b; Jia et al., 2011; Zhao et al., 2011b; Yang et al., 2018), [As_3_Q_6_]^3-^ (An et al., 2017; Fu et al., 2005a; Zhou et al., 2017), [As_4_Q_6_]^2-^ (Ansari et al., 1992; Smith et al., 1996), [As_4_S_7_]^2-^ (Vater and Sheldrick, 1997), [As_4_Q_8_]^4-^ (Kromm and Sheldrick, 2008), [As_6_S_10_]^2-^ (Vater and Sheldrick, 1997), [As_8_S_13_]^2-^ (Sheldrick and Kaub, 1985b; Sheldrick and Kaub, 1985a; Vater and Sheldrick, 1998), [As_10_Q_3_]^2-^ (Smith et al., 1996). The thiophilic metal ions (Cu^+^, Ag^+^, Cd^2+^, Hg^2+^), which have less or little tendency to form complex cations with the strongly chelating amines, usually bond directly to the chalcogen elements. So far, some [M_x_As_y_Q_z_] chalcogenidoarsenates (M = Cu, Ag, Cd, Hg) have been obtained under solvothermal conditions. Such as, two kinds of [Cu_2_AsS_3_]_n_^−^ chains (5-membered Cu_2_AsS_2_ rings and 6-membered Cu_2_AsS_3_ rings) form the two-dimensional anionic [Cu_2_AsS_3_]_n_^−^ layer (Yao et al., 2013). The one-dimensional [AgAsS_4_]_n_^2n−^ chain is a result of corner and edge sharing between AgS_4_ and AsS_4_. The [AgAs_2_Se_5_]_n_^3-^ chains consist of *ψ*-bitetrahedral [As_2_Se_5_]^4-^ units and tetrahedral coordinated Ag^+^ ions (Wachhold and Kanatzidis, 1999). The three-dimensional [Cu_8_(*μ*_8_-Se) (AsSe_4_)_6/2_]_n_^3-^ framework is constructed of icosahedral Cu_8_Se_13_ clusters linked by As^5+^, with counterions located in the cavities (Zhang et al., 2012). The [CdAs_2_Se_4_]_n_^2-^ chain synthesized by our group is made up of a tetrahedral [CdSe_4_] connected to a dimer As_2_^4+^ through an As-Se bond, whereas the [HgAs_2_Se_4_]_n_^2-^ chain is formed by of a tetrahedral [HgSe_4_] connected to a dimer As_2_^4+^ dimer (Du et al., 2019; Teri et al., 2021b).

However, the addition of transition metals (Fe, Co, Ni, Zn, and Mn) is the most efficient and attractive way to synthesize new classes of chalcogenidoarsenates because these elements possess certain optical, magnetic, and electronic properties (Smith et al., 1996; Jia et al., 2011; Zhou, 2016). In the presence of strongly chelated amines, above transition metal ions are easily able to form stable transition metal complexes with organic amines due to variable coordinating environments. The addition of transition metal cations to the reaction mixture may increase the structural variability and tailor the electronic properties. Transition metal complexes (TMCs) can also act as structural directing agents or charge compensating ions, as exemplified by [M(dien)_2_][As_2_Se_6_] (M = Co, Ni), [Ni(en)_3_]_2_[As_2_S_5_] (en = ethylenediamine), [Fe(phen)_3_][As_2_Se_6_], [Zn(phen) (dien)][As_2_Se_6_]·2phen, [Ni(phen)_3_][As_2_Se_2_(*μ*-Se_3_) (*μ*-Se_5_)] (phen = 1,10-phenanthroline) (Jia et al., 2006; Jia et al., 2011; Zhao et al., 2011b). Noteworthy is that Mn^2+^ ions can not only coordinate with chalcogen atoms, but can also form transition metal complexes, which can connect with the chalcogenidoarsenate framework to generate new organic hybrid chalcogenidoarsenates, and most of these are zero-or one-dimensional, as exemplified by zero-dimensional {[Mn(phen)]_2_(As^V^S_4_)_2_}^2−^ (Liu et al., 2012), {[Mn(dien)]_2_(As^V^S_4_)_2_}^2−^ (Zhou et al., 2015), [Mn_2_(AsS_4_)_4_]^8−^ (Iyer and Kanatzidis, 2004), and one-dimensional {[Mn(phen)]_3_(As^V^S_4_) (As^III^S_3_)}_n_ (Liu et al., 2011), [Mn(teta) (As^V^S_4_)]_n_^−^ (Zhou et al., 2015), [Mn(dien) (AsS_4_)]_n_^n−^ (Fu et al., 2005b), [Mn(en)_2_CuAs^V^S_4_]_n_ (Zhou et al., 2015).

On the basis of these findings, a variety of transition metal complexes were selected as structure-directing agents for the synthesis of selenoarsenates with different structures: [Co(tren)_2_H]AsSe_4_ (tren = tris(2-aminoethyl)amine) (**1**); [Ni_2_(dien)_4_][As_2_Se_5_] (dien = diethylenetriamine) (**2**); [Zn(tren)]_2_[As_2_Se_5_] (**3**) and [Mn(tren)]_2_[As_2_Se_5_] (**4**). According to our knowledge, [Zn(tren)]_2_[As_2_Se_5_] (**3**) is the first [As_2_Se_5_]^4−^ anion type of zinc selenoarsenate. There are two ligand environments of Mn^2+^ ions in [Mn(tren)]_2_[As_2_Se_5_] (**4**), which provides novel selenoarsenate of the [As_2_Se_5_]^4−^ anion type. Adding K_2_CO_3_/Cs_2_CO_3_ to the synthesis system is necessary and may act as a mineralizer. Meanwhile, the pH of the solution also influences their structure. Additionally, their synthesis, structure, physical properties, photocurrent response, and magnetic are described in detail.

## Experimental Section

All raw materials were purchased from the Shanghai Macklin Co., Ltd.: K_2_CO_3_ (99.5%), Cs_2_CO_3_ (99.5%), CoCl_2_·6H_2_O (98.0%), NiCl_2_·6H_2_O (99.0%), Zn(Ac)_2_·2H_2_O (99.0%), MnCl_2_·4H_2_O (99.0%), As_2_S_3_ (99.9%), Se (99.0%), tren (tren = tris(2-aminoethyl)amine) (96.0%), dien (dien = diethylenetriamine) (99.5%), CH_3_OH (99.5%), C_2_H_5_OH (99.7%), PEG-400 (poly-(propylene glycol)-400) (99.5%).

### Synthesis of [Co(tren)_2_H]AsSe_4_ (1)

Cs_2_CO_3_ (17.0 mg, 0.052 mmol), CoCl_2_·6H_2_O (24.0 mg, 0.077 mmol), As_2_S_3_ (12.0 mg, 0.049 mmol) and Se (18.0 mg, 0.228 mmol), and a mixed solvent of tren (500 mg, 3.424 mmol) and C_2_H_5_OH (250 mg, 5.434 mmol) were added to Pyrex glass tube. The glass tube was sealed with a 10% filling, placed into a Teflon-lined stainless steel autoclave and heated at 150°C for 7 d. The products were washed with ethanol and deionized water, respectively, and dark yellow blocks crystal were obtained (27% yield based Se). Elemental analysis for **1**: C 19.31%, H 4.92%, N 15.05%. Calc.: C 19.38%, H 4.97%, N 15.07%.

### Synthesis of [Ni_2_(dien)_4_][As_2_Se_5_] (2)

NiCl_2_·6H_2_O (24.0 mg, 0.101 mmol), As_2_S_3_ (12.0 mg, 0.049 mmol), Se (16.0 mg, 0.203 mmol), and a mixed solvent of dien (630 mg, 4.315 mmol) and CH_3_OH (240 mg, 7.490 mmol) were added to Pyrex glass tube. The glass tube was sealed with a 10% filling, placed into a Teflon-lined stainless steel autoclave and heated at 150°C for 7 d. The products were washed with ethanol and deionized water, respectively, and red blocks crystal were obtained (29% yield based Se). Elemental analysis for 2: C 17.84%, H 4.79%, N 15.58%. Calc.: C 17.90%, H 4.84%, N 15.67%.

### Synthesis of [Zn(tren)]_2_[As_2_Se_5_] (3)

Cs_2_CO_3_ (17.0 mg, 0.052 mmol), Zn(Ac)_2_·2H_2_O (22.0 mg, 0.100 mmol), As_2_S_3_ (12.0 mg, 0.049 mmol), Se (16.0 mg, 0.203 mmol), and a mixed solvent of tren (800 mg, 5.479 mmol) and PEG-400 (250 mg, 4.03 mmol) were added to Pyrex glass tube. The glass tube was sealed with a 10% filling, placed into a Teflon-lined stainless steel autoclave and heated at 160°C for 6 d. The products were washed with ethanol and deionized water, respectively, and yellow blocks crystal were obtained (31% yield based Se). Elemental analysis for 3: C 14.81%, H 3.66%, N 11.52%, calc.: C 14.88%, H 3.71%, N 11.57%.

### Synthesis of [Mn(tren)]_2_[As_2_Se_5_] (4)

K_2_CO_3_ (14.0 mg, 0.111 mmol), MnCl_2_·4H_2_O (12.0 mg, 0.061 mmol), As_2_S_3_ (12.0 mg, 0.049 mmol), Se (16.0 mg, 0.203 mmol), and tren of solvent (600 mg, 4.109 mmol) to a Pyrex glass tube. The glass tube was sealed with a 10% filling, placed into a Teflon-lined stainless steel autoclave and heated at 150°C for 7 d. The products were washed with ethanol and deionized water, respectively, and yellow rodlike crystal were obtained (21% yield based Se). Elemental analysis for **4**: C 15.16%, H 3.77%, N 11.77%, Calc: C 15.21%, H 3.80%, N 11.83%.

## Results and Discussion

### Syntheses

It has been widely observed that transition metal complexes are useful as template or structural-directing agents in the synthesis of chalcogenides. In this work, we have synthesized successfully four novel selenoarsenates in amine-alcohol system by solvothermal method, [Co(tren)_2_H]AsSe_4_ (**1**); [Ni_2_(dien)_4_][As_2_Se_5_] (**2**); [Zn(tren)]_2_[As_2_Se_5_] (**3**) and [Mn(tren)]_2_[As_2_Se_5_] (**4**). When organic amines and second agents (e.g., methanol, ethanol, polyethylene glycol) are used as mixed solvents in the synthesis of compounds **1**, **2** and **3**, their effects on crystallization are favorable. Conversely, when the second agent was not present, there was a significant loss of yield. In all probability, this is due to the drastic changes in some physical properties of the solvent (e.g., pH, density, viscosity, and diffusion coefficient), which contribute to the increased solubility and diffusivity of the reactants, as well as crystal growth. Furthermore, we found that adding K_2_CO_3_/Cs_2_CO_3_ was necessary for the synthesis of compounds **1**, **3** and **4**. If K_2_CO_3_/Cs_2_CO_3_ is removed from the reaction system, then the target product is not obtained, indicating its role as a mineralizer. The mineralizer may not be involved in the crystal structure but is crucial to the preparation of chalcogenides.

### Structural Descriptions

**FIGURE 1:**
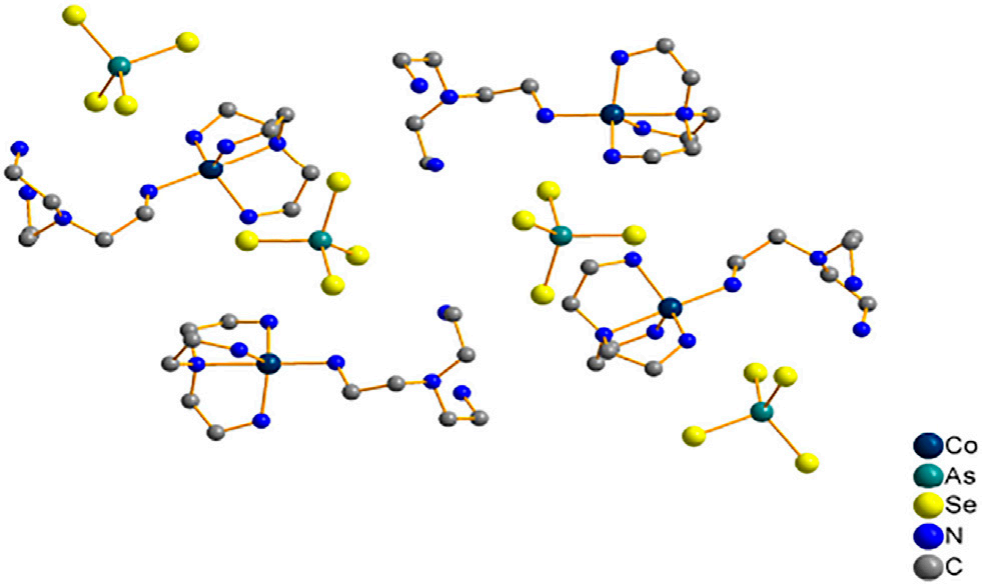
Crystal structure of compound **1** (hydrogen atoms are omitted for clarity).

Compound **1** crystallizes in the monoclinic crystal system in space group *P*2_1_/*n,* and it consist of discrete tetrahedral AsSe_4_ and trigonal-bipyramid [Co(tren)_2_]^2+^ (Figure 1). The arsenic atoms have the pentavalent state, and they form tetrahedra AsSe_4_ by bonding with four Se atoms. Co^2+^ coordinates with four N atoms of one tren ligand and one N atom of another tren ligand to form a trigonal-bipyramid [Co(tren)_2_]^2+^ complex cation. In the compound [Co(phen)_3_][As_2_Se_2_(*μ*-Se_3_) (*μ*-Se_5_)] (Zhao et al., 2011a), however, arsenic atoms adopt a trivalent state. And the [As_2_Se_2_(*μ*-Se_3_) (*μ*-Se_5_)]^2−^ anion contains two crystallographically As^3+^ centres, and each is coordinated by a terminal Se^2−^ to give AsSe^+^ units. The AsSe^+^ units are joined together by *μ*-Se_3_^2−^ and *μ*-Se_5_^2−^ bridging ligands to give rise to a one-dimensional chain [As_2_Se_2_(*μ*-Se_3_) (*μ*-Se_5_)]^2−^ (Du et al., 2019). Moreover, in the compound [Co(peha)][Co(As_3_S_3_)_2_] (Han et al., 2016), the arsenic atom binds three S^2−^ ions, forming a typical trigonal pyramid AsS_3_. The adjacent trigonal pyramid AsS_3_ unit is connected by an arsenic atom via two S^2−^ ions, forming As_3_S_3_ aggregation. Also the two As_3_S_3_ aggregates coordinate to the Co^2+^ ion via two arsenic atoms and an S atom to form [Co(As_3_S_3_)_2_]_2_ cluster. For compound **1**, the As-Se bonds range between 2.2679(13) and 2.2932(11) Å, while Se-As-Se bond angles range from 106.74(5) to 112.48(5) ^°^. The Co-N bond length ranges from 2.057(8) to 2.258(6) Å with N-Co-N bond angles from 79.3(3) to 178.4(2) ^°^. The bond lengths and angles reported here are similar to those reported previously (Zhao et al., 2011a; Han et al., 2016).

**FIGURE 2:**
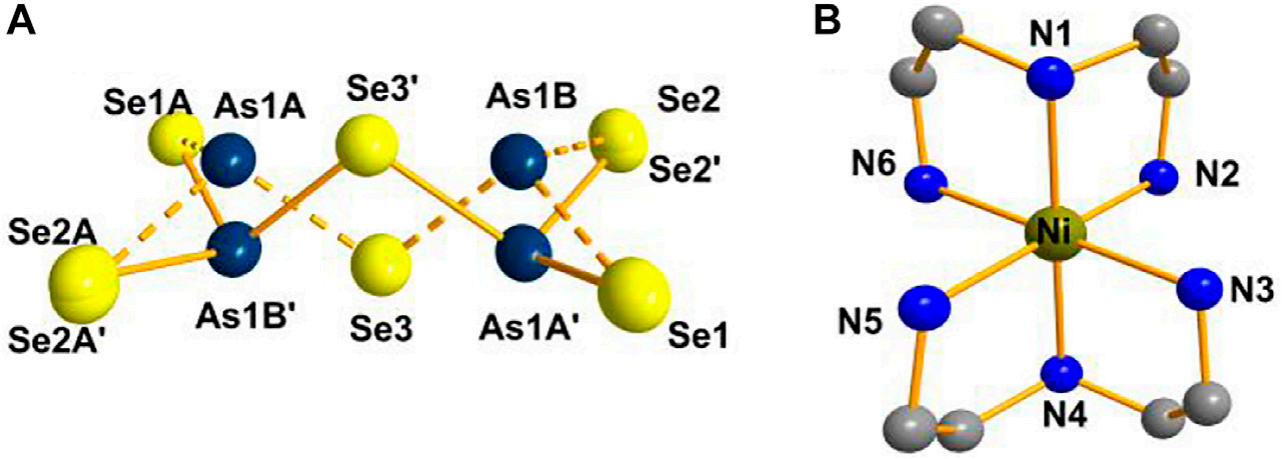
**(A)** [As_2_Se_5_]^4-^ anion. **(B)** Coordination environment of the Ni^2+^ ion in compound **2** (hydrogen atoms are omitted for clarity).

Compound **2** crystallizes in the monoclinic crystal system in space group *P*2_1_/*n*. Two [AsSe_3_] trigonal pyramids are joined via corner sharing to form the [As_2_Se_5_]^4−^ anion (Figure 2A). The As(1), Se(2) and Se(3) atoms are disordered. As shown in Figure 2B, the Ni^2+^ ion is coordinated by six N atoms to produce octahedral [Ni(dien)_2_]^2+^. Supplementary Figure S1 shows the cluster structure of the compound **2**. Unlike the compound **2**, [Ni(en)_3_]_2_[As_2_S_5_] contains arsenic atoms that adopt a pyramidal coordination geometry by bonding with three S atoms to form the [AsS_3_] pyramid. Two [AsS_3_] pyramids form dimeric [As_2_S_5_]^4−^ anion by corner-sharing, and the two AsS_3_ pyramids are in *cis*-conformation (Jia et al., 2006). For compound **2**, the As-Se bonds range from 1.8755(18) to 2.4404(19) Å and Se-As-Se bond angles range from 52.91(8) to 133.15(8)^°^. The Ni-N bond lengths range from 2.081(6) to 2.135(6) Å while the N-Ni-N bond angles range from 82.2(2) to 178.9(2)^°^. According to literature, bond lengths and angles are consistent (Jia et al., 2006; Du et al., 2019).

**FIGURE 3:**
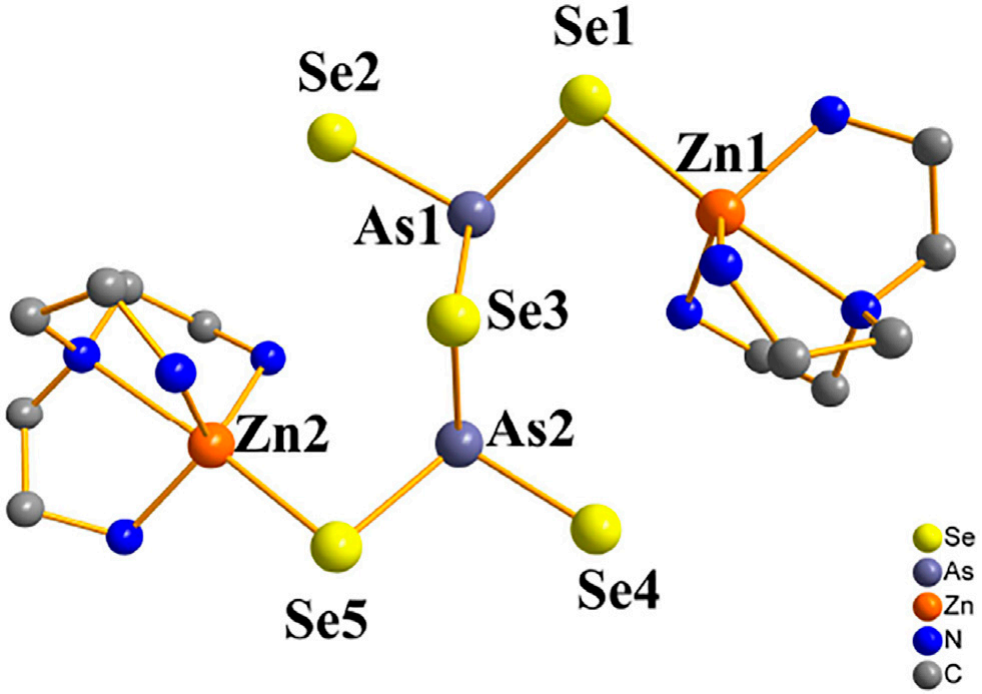
Trigonal-bipyramidal unsaturated [Zn(tren)]^2+^ caiton of compound **3** (hydrogen atoms are omitted for clarity).

Compound **3** crystallizes in the monoclinic crystal system in space group *C*2*/c*. In contrast to compound **1** and **2**, in compound **3** the arsenic atoms are trivalent and they join three Se^2−^ ions to form pyramid AsSe_3_, followed by two trigonal pyramid AsSe_3_ units are linked to *μ*_2_ *-*Se(3) to form dimers [As_2_Se_5_]^4−^. Figure 3 shows the trigonal-bipyramidal unsaturated [Zn(tren)]^2+^ caiton formed by the coordination of the Zn^2+^ ion with four N atoms of the tren ligands and one *μ*_2_ *-*Se atom. And two unsaturated [Zn(tren)]^2+^ caiton forms a cluster structure via dimeric [As_2_Se_5_]^4−^ linkage between them. The cluster structure of compound **3** is shown in Supplementary Figure S2. Comparison with only one [As_2_Se_6_]^2−^ anion type of zinc selenoarsenate [Zn(phen) (dien)][As_2_Se_6_]·2phen is quite different (Jia et al., 2011). In the [Zn(phen) (dien)][As_2_Se_6_]·2phen, the AsSe_3_ pyramids are tied together with two Se-Se bonds, forming the dimeric anion [As_2_Se_6_]^2−^, each [As_2_Se_6_]^2−^ anion contains a six-membered ring. With compound **3**, As-Se bond lengths range from 2.3181(10) to 2.4685(10) Å; Se-As-Se bond angles are between 86.92(3) and 105.74(4) ^°^; and Zn-N bond lengths are between 2.074(6) and 2.416(6) Å; N-Zn-N bond angles are between 76.8(2) and 171.07(14) ^°^. The bond lengths and angles are consistent with those reported in the literature (Jia et al., 2011; (Teri et al., 2021b). To the best of our knowledge, compound **3** is the first zinc selenoarsenate containing [As_2_Se_5_]^4-^ anion type.

**FIGURE 4:**
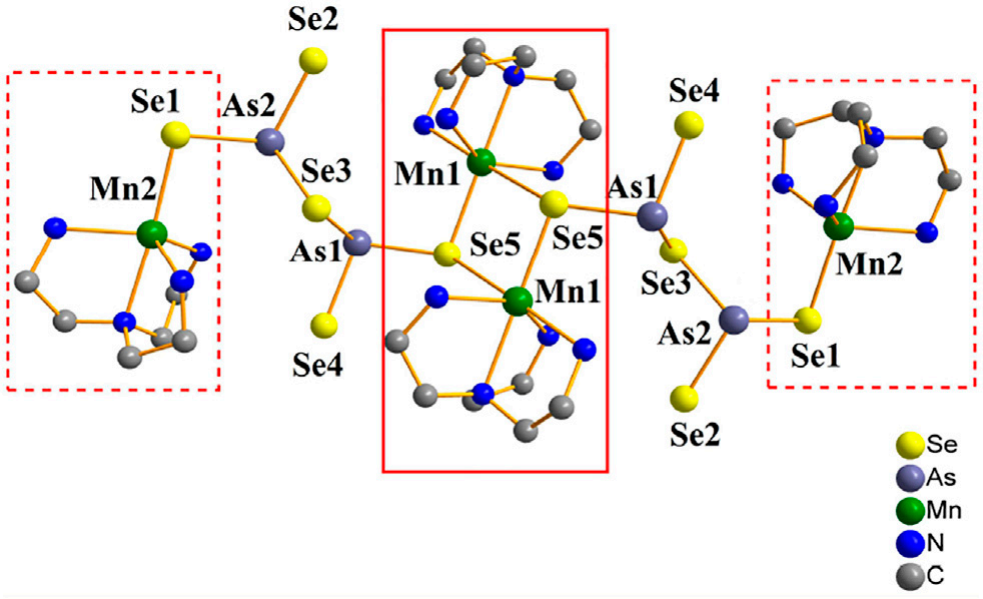
Clusters of [Mn(tren)]_4_[As_4_Se_10_] in compound **4** (hydrogen atoms are omitted for clarity).

Compound **4** crystallizes in the triclinic crystal system in space group *P* $\bar{1}$ . The arsenic atoms in compound **4** are coordinated in the same way as those in compound **3** and have the same dimeric [As_2_Se_5_]^4−^ unit. There are two kinds of coordination modes for Mn^2+^ ions: (1) *μ*_5_ *-*Mn(2)^2+^ ion is coordinated by four N atoms of one tren ligands and one *μ*_2_ *-*Se(1) atom to form trigonal-bipyramidal [Mn(tren)]Se (the [Mn(tren)]Se is outlined by a dashed line area in Figure 4); (2) *μ*_6_ *-*Mn(1)^2+^ ion is coordinated by four N atoms of one tren ligands and two *μ*_3_ *-*Se(5) atoms to formed octahedral [Mn(tren)]Se_2_. Firstly, two octahedra [Mn(tren)]Se_2_ sharing edges, forming a unsaturated [Mn(tren)]^2+^ caiton (the unsaturated [Mn(tren)]^2+^ caiton is outlined by a solid line area). Further, the cluster is connected to two trigone-bipyramidal [Mn(tren)]Se via the dimer [As_2_Se_5_]^4−^ to form [Mn(tren)]_4_[As_4_Se_10_]. The cluster structure of compound **4** is shown in Supplementary Figure S3. When compared to any manganese selenoarsenate of the [As_2_Q_5_]^4−^ anion type, the structures are quite different. As an example, [{Mn(terpy)}_2_(*μ*-As_2_Se_5_)] consists of dipyramidal [As_2_Se_5_]^4−^ ligands that span three Mn^II^ atoms in a tetradentate pattern of *μ*_3_-1*κ* ^2^Se^1^, Se^2^:2*κ*Se^4^: 3*κ*Se^5^. Tetranuclear complexes are centrosymmetric and exhibit an 8-member ring (MnSeAsSe)_2_ (Kromm and Sheldrick, 2008). Nevertheless, in [Mn(en)_3_]_2_As_2_Se_5_, there are isolated anions [As_2_Se_5_]^4−^ and transition metal cations [Mn(en)_3_]^2+^. Initially, the dimeric [As_2_Se_5_]^4−^ anion was isolated and co-crystallized with transition metal complex cations as counterions, composed of two corner-sharing AsSe_3_ trigonal pyramids (Jia et al., 2006). It has been proposed that compound Mn_2_(2,2′-bipy)As_2_^III^S_5_ is formed from four-cubane [Mn_6_(2,2′-bipy)_4_As_6_^III^S_14_]^2+^, which are interlinked by face-sharing to form a two-dimensional network. There are also two coordination environments for the Mn atom. The Mn atom is coordinated by six sulfur atoms from three [As_2_^III^S_5_]^4−^ groups; the Mn atom is chelated by two 2,2-bipy ligands and coordinated by four sulfur atoms from two [As_2_^III^S_5_]^4−^ groups (Fu et al., 2006). In compound **4**, As-Se bonds range from 2.3291(17) to 2.4628(17) Å with Se-As-Se angles between 102.04(6) and 105.29(6) ^°^. Mn-N bond lengths range from 2.192(10) to 2.368(9) Å and N-Mn-N bond angles range from 75.8(4) to 117.8(4) ^°^. According to the published literature, these bond lengths and angles are similar (Fu et al., 2006; Jia et al., 2006). We found that in compound **4**, Mn^2+^ ions had two coordination modes, which was unusual in previous reports.

Compounds **3** and **4** have some special structural features. First, in compounds **3** and **4**, it is the dimeric [As_2_Se_5_]^4−^ unit that is linked to the transition metal complex. To the best of our knowledge, this type of connection mode has never been done before. It appears that most of them are directly linked to transition metal complexes via the AsSe_x_ (x = 3,4) units (Chen et al., 2021; Zhao et al., 2011b). Second, [As_2_Se_6_]^2−^ anion type zinc selenoarsenate was synthesized in 2011 by Jia’s group (Jia et al., 2011). In addition, compound **3** is the first [As_2_Se_5_]^4−^ anion type of zinc selenoarsenate, which lays a foundation for future work. Lastly, in compound **4**, the five- and six-coordinate manganese atoms are linked by dimeric [As_2_Se_5_]^4−^ units to form [Mn(tren)]_4_[As_4_Se_10_] clusters. Therefore, compounds **3** and **4** present a new structural pattern.

### Powder X-Ray Diffraction and Thermogravimetric-Differential Thermal Analysis

In Supplementary Figure S4, the position of the 2*θ* diffraction peak obtained by the experiment is consistent with simulation results of the analysis of a single-crystal structure, showing that the products of the compounds are very pure, and all samples can be used for further study. The thermal stability of compounds **1**-**4** was studied by thermogravimetric and differential thermal analysis (Supplementary Figure S5). During the test, compound **1,** the weight loss rate of 14% in the range of 181–286°C, which was consistent with the loss of one molecule of H_2_Se (theoretical weight loss rate of 10.89%), and at approximately 400–550°C have a significant weight loss rate of 42% (the theoretical weight loss rate of 40.65%), which may be due to the loss of two molecules of tren organic amine, and was accompanied by an endothermic peak at 300 and 578°C in the DTA curve. The weight loss rate of compound **2** is 36% between 346 and 451°C, consistent with the loss of four molecules of dien ligand (theoretical weight loss rate of 38.48%), and the DTA curve shows an endothermic peak at 398°C. Compound **3** has a weight loss of 31% between 252 and 305°C, which is consistent with the loss rate of two molecules of tren ligand (30.22%), and has an endothermic peak at 323°C in the DTA curve. Compound **4** has a weight loss rate of 32% between 200 and 295°C, which is consistent with the loss of a molecule of tren ligand (theoretical weight loss rate of 30.88%). At the same time, the endothermic peak occurs at 249°C on the DTA curve. The appearance of an endothermic peak can be attributed to the formation of amorphous material by structural collapse at a given temperature.

### Infrared Spectra

FT-IR spectrum (Supplementary Figure S6) shows that compounds **1**, **3**, **4** mainly originate from organic solvent tren, and the absorption peaks of compound **2** are mainly due to dien. In the **1**, **3**, **4**, a strong absorption peak between 3,196 cm^−1^ and 3,092 cm^−1^ is due to the stretching vibration of the N-H bond; the strong N-H bending vibration appears separately at 1,567, 1,624, 1,601 cm^−1^; the C-H stretching vibration occurs between 2,968 and 2,838 cm^−1^; the -CH_2_- bending vibration peak individually appears at 1,466, 1,466, 1,452 cm^−1^; C-C, C-N stretching vibration absorption peaks are located in the region of 1,389–1,002 cm^−1^; weak N-H, C-H bending vibration peaks are located in the region of 994–523 cm^−1^. In compound **2**, the absorption peak at 3,196 cm^−1^ and 3,104 cm^−1^ is caused by N-H stretching vibrations. There is a weak C-H stretching vibration absorption peak in the range of 2,911–2,857 cm^−1^; at 1,452 cm^−1^, there is a strong -CH_2_- bending vibration absorption peak; the absorption peaks in the range of 1,384–1,077 cm^−1^ are the result of C-C and C-N stretching vibrations; there are weak N-H and C-H bending vibration absorption peaks in the range of 951–527 cm^−1^.

### Photocurrent Responses

**FIGURE 5:**
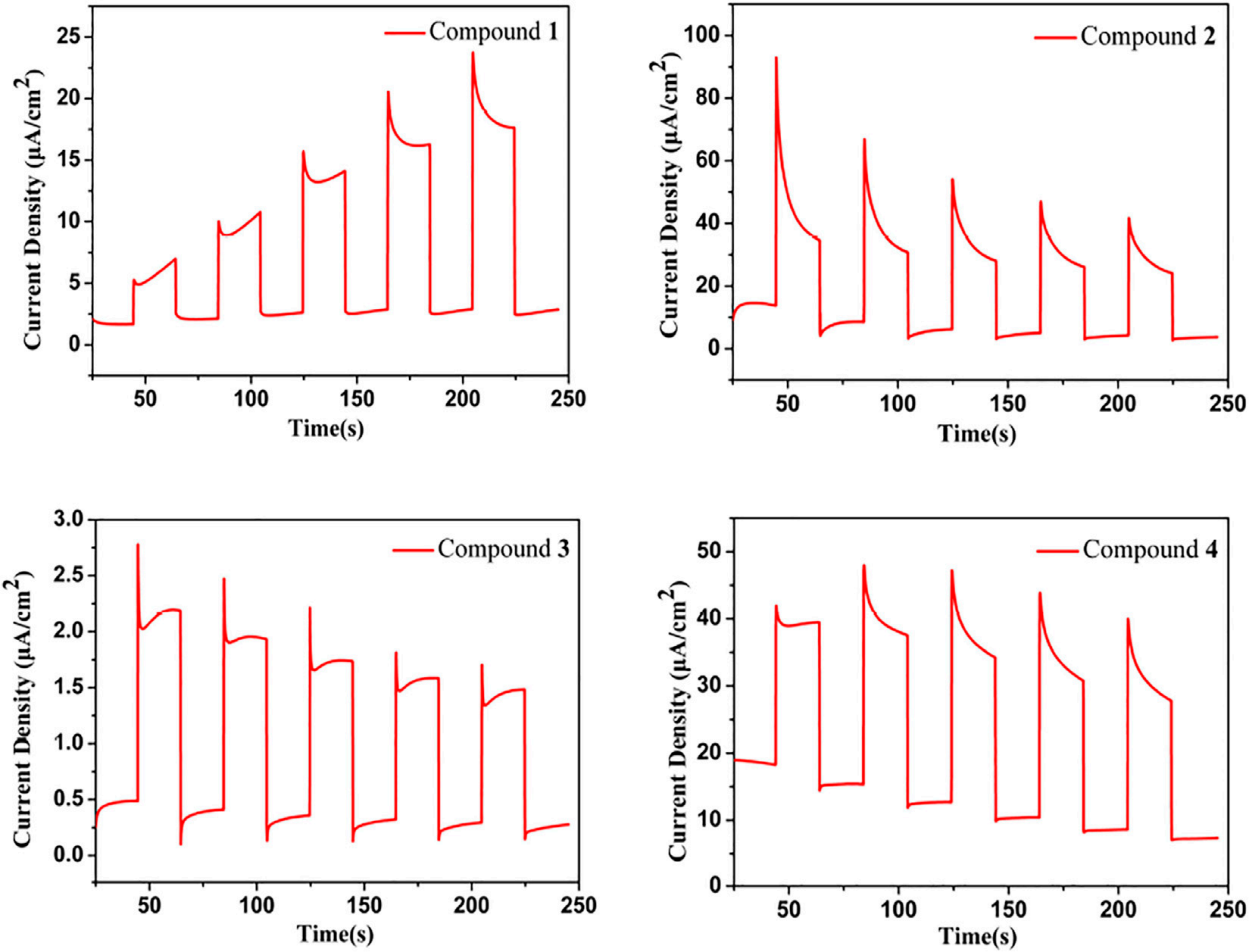
The photocurrent responses of **1–4** (a–d).

Using a 300 W xenon lamp exposed to visible light (*λ* ≥ 420 nm) for photocurrent measurements, repeatable responses were observed for compounds **1**–**4**. Compounds **1**-**4** exhibit good photocurrent profiles under visible light illumination as shown in Figure 5. Compounds **2** and **4** have twice the photocurrent density of **1** and **3**. A high photocurrent indicates that the compound has a high photoelectron transfer efficiency, which provides evidence for the application of their photocatalytic properties. The photocurrent densities of compound **2** and **4** are much higher than those of other chalcogenides, including [pipH_2_]_2_[pipH]_2_[In_2_As^III^_2_As^V^_2_S_10.2_Se_3.1_(Se_2_)_0.7_] (ca. 47 nA/cm^2^) (Wang et al., 2020), Rb_2_Ba_3_Cu_2_Sb_2_S_10_ (ca. 6 nA/cm^2^) (Liu et al., 2020), K_3_Mn_2_Sb_3_S_8_ (ca. 6 nA/cm^2^) (Xiao et al., 2021), K_2_HgSnSe_4_ (ca. 3 μA/cm^2^) (Teri et al., 2021a), Cs_2_Ag_6_As_2_S_7_ (ca. 5 μA/cm^2^) (Li et al., 2021), and [Zn(tren)_2_H]SbSe_4_ (ca. 10 μA/cm^2^) (Shele et al., 2021).

### Magnetic Properties

**FIGURE 6:**
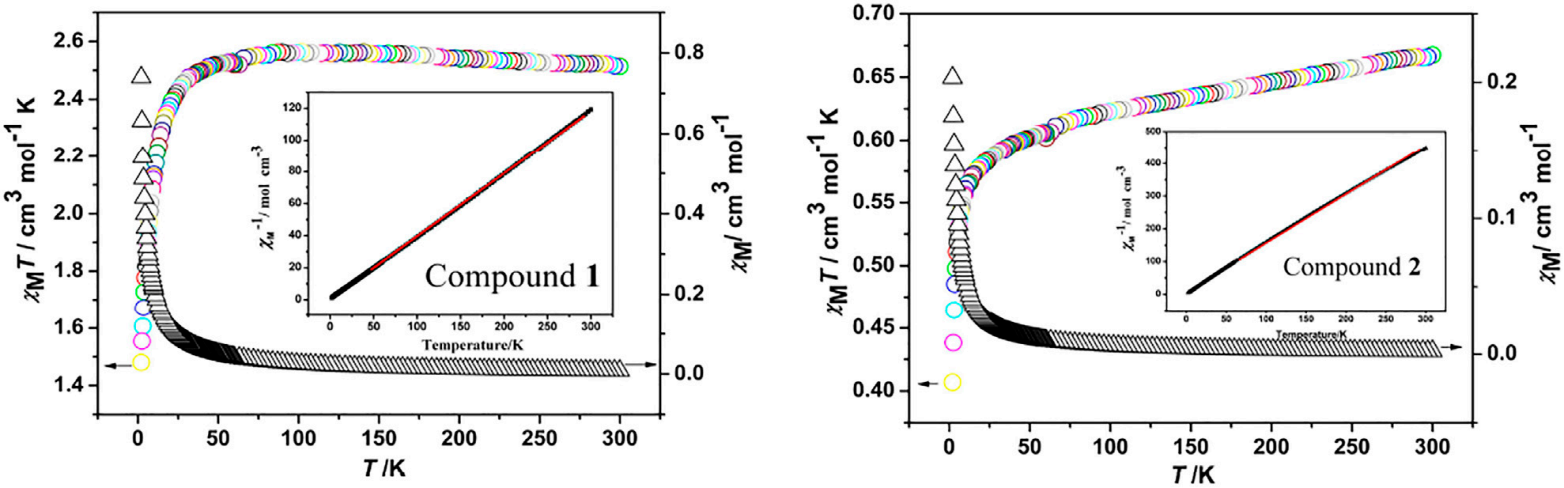
Temperature dependence of χ_M_ and χ_M_T for compounds. Insert: the temperature dependence of 1/χ_M_ for compounds with the solid line representing the fit of the Curie-Weiss law.

The variable-temperature magnetic susceptibility data were collected for compounds at an applied dc field of 1,000 Oe in the 2–300 K temperature range. The *χ*_M_ *T vs T* and *χ*_M_ *vs. T* plots for **1** and **2** are shown in Figure 6. For compound **1**, the *χ*_M_ *T* value at 300 K is 2.51 cm^3^ mol^−1^ K, and after cooling, the *χ*_M_ *T* value falls to 1.48 cm^3^ mol^−1^ K at 2 K. Meanwhile, *χ*_M_ gradually increases from 0.008 cm^3^ mol^−1^ at 300 K to a value of 0.739 cm^3^ mol^−1^ at about 2 K. This character suggests the dominant antiferromagnetic interaction between the Co^II^ centers in **1**. The 1/*χ*_M_ *vs T* curve above 50 K obeys the Curie-Weiss law with *C* = 2.54 cm^3^ mol^−1^ K and *θ* = -0.65 K (Figure 6, insert). The negative *θ* value further confirms the antiferromagnetic coupling among the Co^2+^ ions. In compound **2**, reached a maximum *χ*_M_ *T* value of 0.66 cm^3^ mol^−1^ K at 300 K and a minimum value of 0.40 cm^3^ mol^−1^ K at 2 K as temperature decreased. Furthermore, *χ*_M_ gradually increases from 0.002 cm^3^ mol^−1^ at 300 K to a value of 0.203 cm^3^ mol^−1^ at about 2 K. This character suggests the dominant antiferromagnetic interaction between the Ni^II^ centers in **2**. The 1/*χ*_M_ *vs T* curve above 60 K obeys the Curie-Weiss law with *C* = 0.56 cm^3^ mol^−1^ K and *θ* = -4.45 K. The negative *θ* value further confirms the antiferromagnetic coupling among the Ni^2+^ ions.

## Conclusion

As a summary, we have synthesized four selenoarsenates using different transition metal complexes as structure-directing agents. **1** has discrete tetrahedral AsSe_4_ in the presence of the transition metal complex [Co(tren)_2_]^2+^. **2** contains the transition metal complex [Ni(dien)_2_]^2+^ and [As_2_Se_5_]^4-^ cluster. In **3**, the transition metal complex unsaturated [Zn(tren)]^2+^ caiton is directly linked to the dimer [As_2_Se_5_]^4-^. Interestingly, the transition metal complexes of two different coordination modes of Mn^2+^ are connected through the dimer [As_2_Se_5_]^4-^ in **4**. Thus, different transition metal complexes that act as structure-directing agents have a significant effect on the structure of selenoarsenates, resulting in fundamentally different structures for **1**–**4**. The photoelectrochemical tests show the compounds have good photocurrent response properties. A study of their magnetic properties has been conducted as well. In addition to providing insight into the structure of chalcogenidoarsenates, the work provided potential applications in optoelectronics.

## Data Availability

The original contributions presented in the study are publicly available. This data can be found here: https://www.ccdc.cam.ac.uk/, 2062155, 2062157, 2040069, 2062154. CCDC numbers 2062155 for 1, 2062157 for 2, 2040069 for 3 and 2062154 for 4 contain the supplementary crystallographic data for this paper. These data can be obtained free of charge via http://www.ccdc.cam.ac.uk/conts/retrieving.html, or from the Cambridge Crystallographic Data Centre, 12 Union Road.

## Supplementary Material

Supplementary Data Sheets 1–6 Cluster structures, physical measurements, including powder X-ray diffraction (PXRD), elemental analysis, electrochemical characterizations, Thermogravimetric-Differential thermal analysis (TG-DTA) and infrared (IR), magnetic susceptibility measurement and tables of crystallographic data.
