# Supplementary material for: Strong Photocurrent Response of Selenoarsenates With Different Transition Metal Complexes as Structure-Directing Agents
Source: Front Chem. 2022 May 5;10:890496. doi: 10.3389/fchem.2022.890496 (PMC9117718; doi:10.3389/fchem.2022.890496)

checkCIF/PLATON report

Structure factors have been supplied for datablock(s) 2

THIS REPORT IS FOR GUIDANCE ONLY. IF USED AS PART OF A REVIEW PROCEDURE FOR PUBLICATION, IT SHOULD NOT REPLACE THE EXPERTISE OF AN EXPERIENCED CRYSTALLOGRAPHIC REFEREE.

No syntax errors found. CIF dictionary Interpreting this report

Datablock: 2

```
Bond precision:   C-C = 0.0105 A          Wavelength=0.71073

Cell:             a=9.4198(4)     b=14.3828(5)        c=12.6237(4)
                  alpha=90        beta=91.500(3)      gamma=90
Temperature:      293 K

                 Calculated                    Reported
Volume           1709.71(11)                   1709.71(11)
Space group      P 21/n                        P 1 21/n 1
Hall group       -P 2yn                        -P 2yn
Moiety formula   2(C8 H25 N6 Ni), As2 Se5      As2 Se5, 2(C8 H25 N6 Ni)
Sum formula      C16 H50 As2 N12 Ni2 Se5       C16 H50 As2 N12 Ni2 Se5
Mr               1072.70                       1072.74
Dx,g cm-3        2.084                         2.084
Z                2                             2
Mu (mm-1)        8.373                         8.373
F000             1044.0                        1044.0
F000'            1045.07
h,k,lmax         12,19,17                      11,19,17
Nref             4560                          3996
Tmin,Tmax        0.311,0.605                   0.400,1.000
Tmin'            0.100

Correction method= # Reported T Limits: Tmin=0.400 Tmax=1.000
AbsCorr = MULTI-SCAN

Data completeness= 0.876           Theta(max)= 29.021

R(reflections)= 0.0509( 2888)                          wR2(reflections)=
                                                       0.1323( 3996)
S = 1.050                  Npar= 199
```

The following ALERTS were generated. Each ALERT has the format
test-name_ALERT_alert-type_alert-level.
Click on the hyperlinks for more details of the test.

Alert level C

```
PLAT094_ALERT_2_C Ratio of Maximum / Minimum Residual Density ....       2.25 Report
PLAT221_ALERT_2_C Solv./Anion  Resd 2 Se   Ueq(max)/Ueq(min) Range        4.7 Ratio
PLAT250_ALERT_2_C Large U3/U1 Ratio for Average U(i,j) Tensor ....        2.1 Note
PLAT324_ALERT_2_C Check for Possibly Missing H on Coordinating....         N1 Check
PLAT341_ALERT_3_C Low Bond Precision on  C-C Bonds ..............     0.0105 Ang.
PLAT420_ALERT_2_C D-H Bond Without Acceptor  N2       --H2A      .     Please Check
PLAT420_ALERT_2_C D-H Bond Without Acceptor  N3       --H3A      .     Please Check
PLAT906_ALERT_3_C Large K Value in the Analysis of Variance ......      2.996 Check
PLAT910_ALERT_3_C Missing # of FCF Reflection(s) Below Theta(Min).          8 Note
PLAT971_ALERT_2_C Check Calcd Resid. Dens.  1.69Ang From As1             2.23 eA-3
PLAT971_ALERT_2_C Check Calcd Resid. Dens.  0.75Ang From As2             1.65 eA-3
PLAT975_ALERT_2_C Check Calcd Resid. Dens.  0.99Ang From N1      .       0.46 eA-3
```

Alert level G

```
PLAT007_ALERT_5_G Number of Unrefined Donor-H Atoms .............           9 Report
PLAT042_ALERT_1_G Calc. and Reported Moiety Formula Strings Differ     Please Check
PLAT199_ALERT_1_G Reported _cell_measurement_temperature ..... (K)         293 Check
PLAT200_ALERT_1_G Reported   _diffrn_ambient_temperature ..... (K)         293 Check
PLAT300_ALERT_4_G Atom Site Occupancy of Se1       Constrained at          0.5 Check
PLAT300_ALERT_4_G Atom Site Occupancy of Se2       Constrained at          0.5 Check
PLAT300_ALERT_4_G Atom Site Occupancy of Se3       Constrained at          0.5 Check
PLAT300_ALERT_4_G Atom Site Occupancy of Se4       Constrained at          0.5 Check
PLAT300_ALERT_4_G Atom Site Occupancy of Se5       Constrained at          0.5 Check
PLAT300_ALERT_4_G Atom Site Occupancy of As1       Constrained at          0.5 Check
PLAT300_ALERT_4_G Atom Site Occupancy of As2       Constrained at          0.5 Check
PLAT302_ALERT_4_G Anion/Solvent/Minor-Residue Disorder (Resd  2  )        100% Note
PLAT304_ALERT_4_G Non-Integer Number of Atoms in ..... (Resd  2  )        3.50 Check
PLAT789_ALERT_4_G Atoms with Negative _atom_site_disorder_group  #           7 Check
PLAT794_ALERT_5_G Tentative Bond Valency for Ni1       (II)      .        1.98 Info
PLAT912_ALERT_4_G Missing # of FCF Reflections Above STh/L=  0.600         488 Note
PLAT941_ALERT_3_G Average HKL Measurement Multiplicity ..........          2.7 Low
PLAT978_ALERT_2_G Number C-C Bonds with Positive Residual Density.           0 Info
```

0 **ALERT level A** = Most likely a serious problem - resolve or explain
0 **ALERT level B** = A potentially serious problem, consider carefully
12 **ALERT level C** = Check. Ensure it is not caused by an omission or oversight
18 **ALERT level G** = General information/check it is not something unexpected

3 ALERT type 1 CIF construction/syntax error, inconsistent or missing data
10 ALERT type 2 Indicator that the structure model may be wrong or deficient
4 ALERT type 3 Indicator that the structure quality may be low
11 ALERT type 4 Improvement, methodology, query or suggestion
2 ALERT type 5 Informative message, check

It is advisable to attempt to resolve as many as possible of the alerts in all categories. Often the minor alerts point to easily fixed oversights, errors and omissions in your CIF or refinement strategy, so attention to these fine details can be worthwhile. In order to resolve some of the more serious problems it may be necessary to carry out additional measurements or structure refinements. However, the purpose of your study may justify the reported deviations and the more serious of these should normally be commented upon in the discussion or experimental section of a paper or in the "special_details" fields of the CIF. checkCIF was carefully designed to identify outliers and unusual parameters, but every test has its limitations and alerts that are not important in a particular case may appear. Conversely, the absence of alerts does not guarantee there are no aspects of the results needing attention. It is up to the individual to critically assess their own results and, if necessary, seek expert advice.

Publication of your CIF in IUCr journals

A basic structural check has been run on your CIF. These basic checks will be run on all CIFs submitted for publication in IUCr journals (*Acta Crystallographica*, *Journal of Applied Crystallography*, *Journal of Synchrotron Radiation*); however, if you intend to submit to *Acta Crystallographica Section C* or *E* or *IUCrData*, you should make sure that full publication checks are run on the final version of your CIF prior to submission.

Publication of your CIF in other journals

Please refer to the *Notes for Authors* of the relevant journal for any special instructions relating to CIF submission.

PLATON version of 20/01/2022; check.def file version of 19/01/2022

Datablock 2 - ellipsoid plot

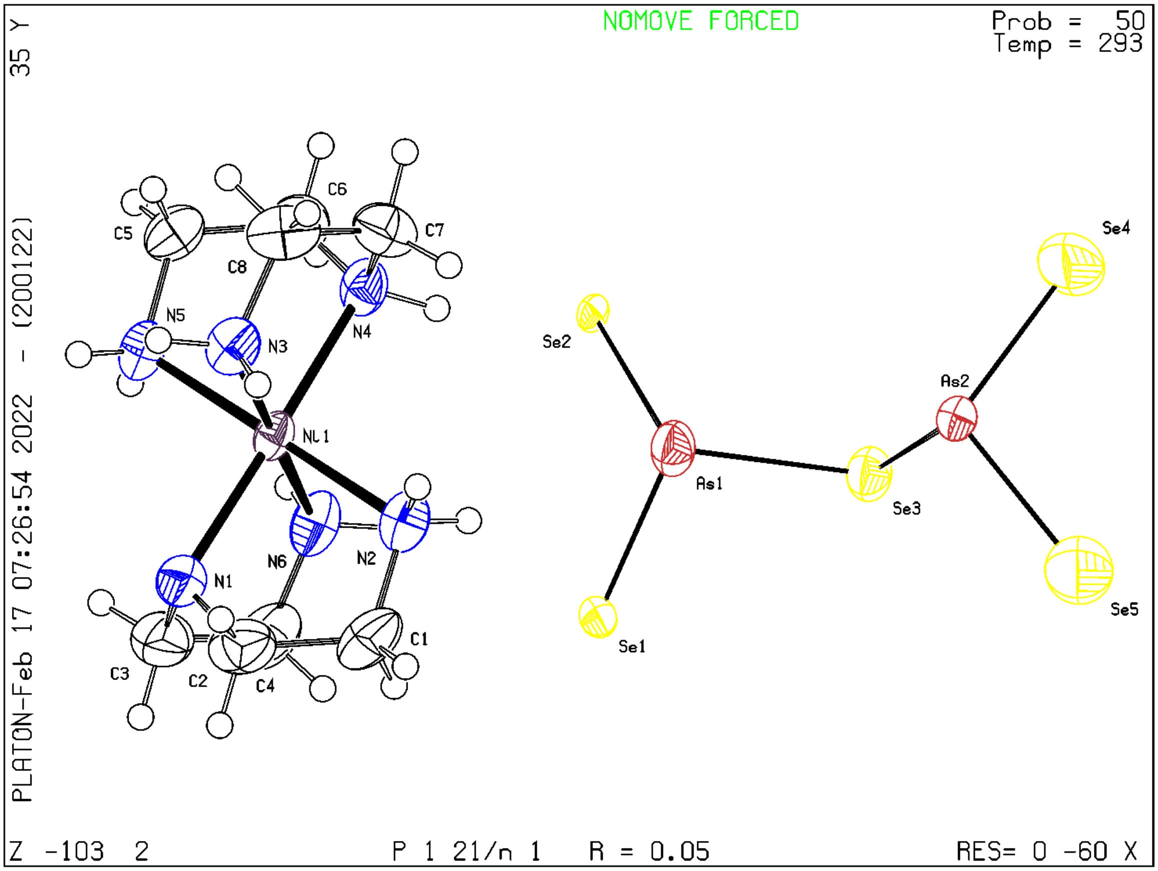

Supplement: Supplementary file 1 [file DataSheet2.PDF]
