# Supplementary material for: Strong Photocurrent Response of Selenoarsenates With Different Transition Metal Complexes as Structure-Directing Agents
Source: Front Chem. 2022 May 5;10:890496. doi: 10.3389/fchem.2022.890496 (PMC9117718; doi:10.3389/fchem.2022.890496)

checkCIF/PLATON report

You have not supplied any structure factors. As a result the full set of tests cannot be run.

THIS REPORT IS FOR GUIDANCE ONLY. IF USED AS PART OF A REVIEW PROCEDURE FOR PUBLICATION, IT SHOULD NOT REPLACE THE EXPERTISE OF AN EXPERIENCED CRYSTALLOGRAPHIC REFEREE.

No syntax errors found. CIF dictionary Interpreting this report

Datablock: 11

```
Bond precision:  C-C = 0.0185 A          Wavelength=0.71073

Cell:           a=8.2043(4)       b=12.0848(7)     c=14.3493(8)
                alpha=78.421(5)   beta=85.370(4)   gamma=82.026(4)
Temperature:    273 K

                 Calculated                Reported
Volume           1378.21(13)               1378.21(13)
Space group      P -1                      P -1
Hall group       -P 1                      -P 1
Moiety formula   C24 H72 As4 Mn4 N16 Se10  C12 H36 As2 Mn2 N8 Se5
Sum formula      C24 H72 As4 Mn4 N16 Se10  C12 H36 As2 Mn2 N8 Se5
Mr               1894.02                   947.01
Dx,g cm-3        2.282                     2.282
Z                1                         2
Mu (mm-1)        9.918                     9.918
F000             900.0                     900.0
F000'            901.09
h,k,lmax         11,16,19                  10,16,18
Nref             7414                      6427
Tmin,Tmax        0.356,0.820               0.326,1.000
Tmin'            0.120

Correction method= # Reported T Limits: Tmin=0.326 Tmax=1.000
AbsCorr = EMPIRICAL

Data completeness= 0.867          Theta(max)= 29.106

R(reflections)= 0.0718( 4301)     wR2(reflections)= 0.2320( 6427)

S = 1.071                   Npar= 262
```

The following ALERTS were generated. Each ALERT has the format
test-name_ALERT_alert-type_alert-level.
Click on the hyperlinks for more details of the test.

Alert level B

```
DIFMN02_ALERT_2_B  The minimum difference density is < -0.1*ZMAX*1.00
            _refine_diff_density_min given =     -4.037
            Test value =     -3.400
PLAT098_ALERT_2_B Large Reported Min.  (Negative) Residual Density      -4.04 eA-3
PLAT230_ALERT_2_B Hirshfeld Test Diff for    Se1      --As2      .        9.5 s.u.
PLAT230_ALERT_2_B Hirshfeld Test Diff for    Se5      --As1      .       24.4 s.u.
PLAT341_ALERT_3_B Low Bond Precision on  C-C Bonds ...............     0.0185 Ang.
```

Alert level C

```
ABSTY02_ALERT_1_C  An _exptl_absorpt_correction_type has been given without
            a literature citation. This should be contained in the
            _exptl_absorpt_process_details field.
            Absorption correction given as empirical
DIFMN03_ALERT_1_C  The minimum difference density is < -0.1*ZMAX*0.75
            The relevant atom site should be identified.
PLAT213_ALERT_2_C Atom C2              has ADP max/min Ratio .....        3.2 prolat
PLAT241_ALERT_2_C High   'MainMol' Ueq as Compared to Neighbors of        Se5 Check
PLAT241_ALERT_2_C High   'MainMol' Ueq as Compared to Neighbors of         C2 Check
PLAT241_ALERT_2_C High   'MainMol' Ueq as Compared to Neighbors of         C3 Check
PLAT241_ALERT_2_C High   'MainMol' Ueq as Compared to Neighbors of         C5 Check
PLAT242_ALERT_2_C Low    'MainMol' Ueq as Compared to Neighbors of        As1 Check
PLAT242_ALERT_2_C Low    'MainMol' Ueq as Compared to Neighbors of         N2 Check
PLAT420_ALERT_2_C D-H Bond Without Acceptor  N1       --H1A      .     Please Check
PLAT420_ALERT_2_C D-H Bond Without Acceptor  N8       --H8A      .     Please Check
PLAT420_ALERT_2_C D-H Bond Without Acceptor  N8       --H8B      .     Please Check
```

Alert level G

```
PLAT007_ALERT_5_G Number of Unrefined Donor-H Atoms ..............         12 Report
PLAT012_ALERT_1_G No       _shelx_res_checksum Found in CIF ......     Please Check
PLAT042_ALERT_1_G Calc. and Reported Moiety Formula Strings Differ     Please Check
PLAT045_ALERT_1_G Calculated and Reported Z Differ by a Factor ...       0.50 Check
PLAT072_ALERT_2_G SHELXL First  Parameter in WGHT  Unusually Large       0.10 Report
PLAT083_ALERT_2_G SHELXL Second Parameter in WGHT  Unusually Large      13.04 Why ?
PLAT199_ALERT_1_G Reported _cell_measurement_temperature ..... (K)        273 Check
PLAT200_ALERT_1_G Reported   _diffrn_ambient_temperature ..... (K)        273 Check
PLAT232_ALERT_2_G Hirshfeld Test Diff (M-X)  Se5      --Mn1      .       21.6 s.u.
PLAT232_ALERT_2_G Hirshfeld Test Diff (M-X)  Se5      --Mn1_a    .       10.6 s.u.
PLAT941_ALERT_3_G Average HKL Measurement Multiplicity ...........        2.3 Low
```

```
   0 ALERT level A = Most likely a serious problem - resolve or explain
   5 ALERT level B = A potentially serious problem, consider carefully
  12 ALERT level C = Check. Ensure it is not caused by an omission or oversight
  11 ALERT level G = General information/check it is not something unexpected

   7 ALERT type 1 CIF construction/syntax error, inconsistent or missing data
  18 ALERT type 2 Indicator that the structure model may be wrong or deficient
   2 ALERT type 3 Indicator that the structure quality may be low
   0 ALERT type 4 Improvement, methodology, query or suggestion
   1 ALERT type 5 Informative message, check
```

It is advisable to attempt to resolve as many as possible of the alerts in all categories. Often the minor alerts point to easily fixed oversights, errors and omissions in your CIF or refinement strategy, so attention to these fine details can be worthwhile. In order to resolve some of the more serious problems it may be necessary to carry out additional measurements or structure refinements. However, the purpose of your study may justify the reported deviations and the more serious of these should normally be commented upon in the discussion or experimental section of a paper or in the "special_details" fields of the CIF. checkCIF was carefully designed to identify outliers and unusual parameters, but every test has its limitations and alerts that are not important in a particular case may appear. Conversely, the absence of alerts does not guarantee there are no aspects of the results needing attention. It is up to the individual to critically assess their own results and, if necessary, seek expert advice.

Publication of your CIF in IUCr journals

A basic structural check has been run on your CIF. These basic checks will be run on all CIFs submitted for publication in IUCr journals (*Acta Crystallographica*, *Journal of Applied Crystallography*, *Journal of Synchrotron Radiation*); however, if you intend to submit to *Acta Crystallographica Section C* or *E* or *IUCrData*, you should make sure that full publication checks are run on the final version of your CIF prior to submission.

Publication of your CIF in other journals

Please refer to the *Notes for Authors* of the relevant journal for any special instructions relating to CIF submission.

PLATON version of 13/07/2021; check.def file version of 13/07/2021

Datablock 11 - ellipsoid plot

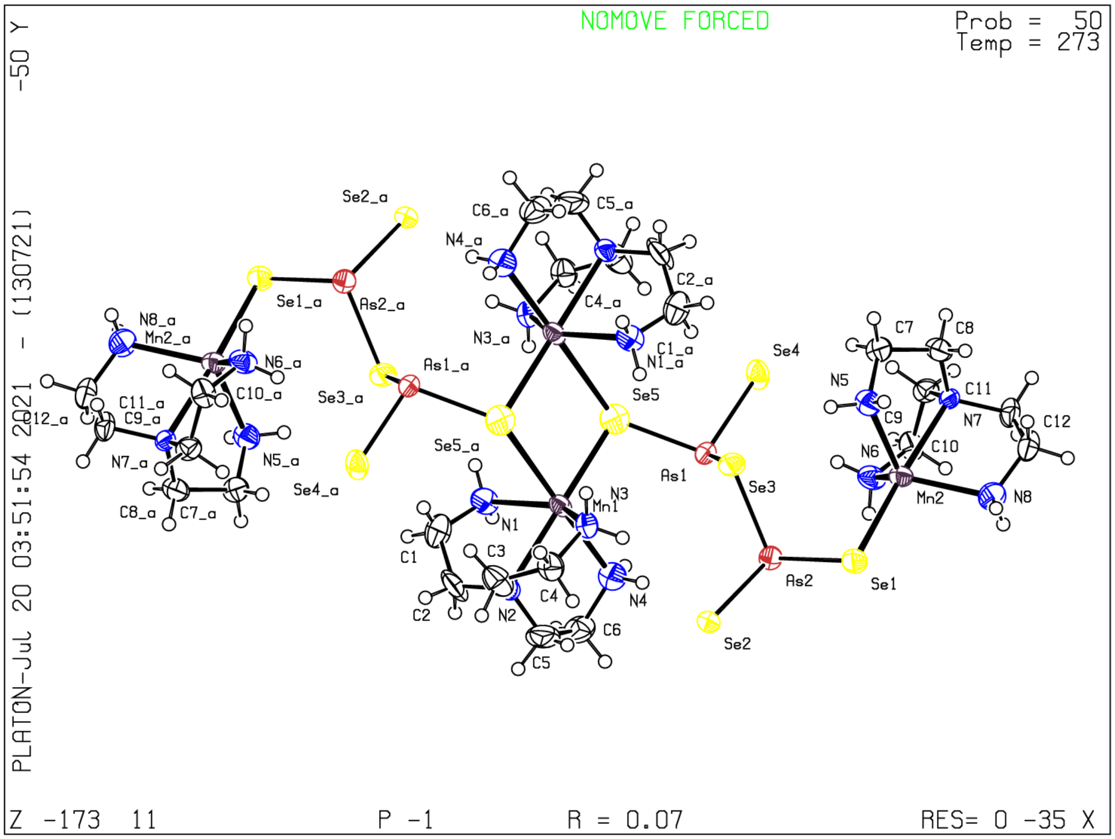

Supplement: Supplementary file 2 [file DataSheet4.PDF]
