# Supplementary material for: Strong Photocurrent Response of Selenoarsenates With Different Transition Metal Complexes as Structure-Directing Agents
Source: Front Chem. 2022 May 5;10:890496. doi: 10.3389/fchem.2022.890496 (PMC9117718; doi:10.3389/fchem.2022.890496)
Supplement: Supplementary file 3 [file DataSheet6.docx]

**Supporting Information**

**Strong Photocurrent Response of Selenoarsenates with Different Transition Metal Complexes as Structure-Directing Agents**

Xinyu Tian^1^, Gele Teri^1^, Muge Shele^1^, Namila E^1^, Liming Qi^1^, Min Liu^1^ and Menghe Baiyin^1^^[[1]](#footnote-1)^*

(College of Chemistry & Environmental Science, Inner Mongolia University Key Laboratory of Advanced Materials Chemistry and Devices (AMC&DLab), Inner Mongolia Normal University, Hohhot, Inner Mongolia 010022, P.R. China)

**Materials and Methods**

The elemental content (C, H and N) of the crystal was measured by Vario Cube elemental analysis meter. The powder X-ray diffraction pattern were collected on a Rigaku XRD-6000 diffractometer under the Cu *Kα* radiation at 40 kV and 40 mA. The patterns were recorded in a slow scanning mode with 2*θ* from 5-80 ° with a scan-rate of 8 °/min. Thermogravimetric-Differential thermal analysis was performed using a Thermal Analysis DTG-60H thermal analyzer under an N_2_ atmosphere. The heating temperature range was 25-800 °C and the heating rate was 10 °C/min. An CHI660E electrochemical workstation was used for photoelectric chemical measurement. A three-electrode system was established using Pt line as counter electrode, Ag/AgCl electrode as reference electrode, ITO working electrode coated with sample and 0.5 M sodium sulfate solution (pH=7.0) as electrolyte. A 300 W Xe lamp (PLS-FX300HU) is used as the light source (*λ*≥420 nm). Infrared spectra were obtained from a powdered sample pelletized with KBr on a Nicolet 6700 in a range of 400-4000 cm^-1^.

**Structure Determination**

For **1-4**, graphite monochromated Mo-*Kα* radiation (*λ*=0.71073 Å) was used on a Bruker APEX-II CCD diffractometer. In addition, all structures were analyzed by using Olex2.


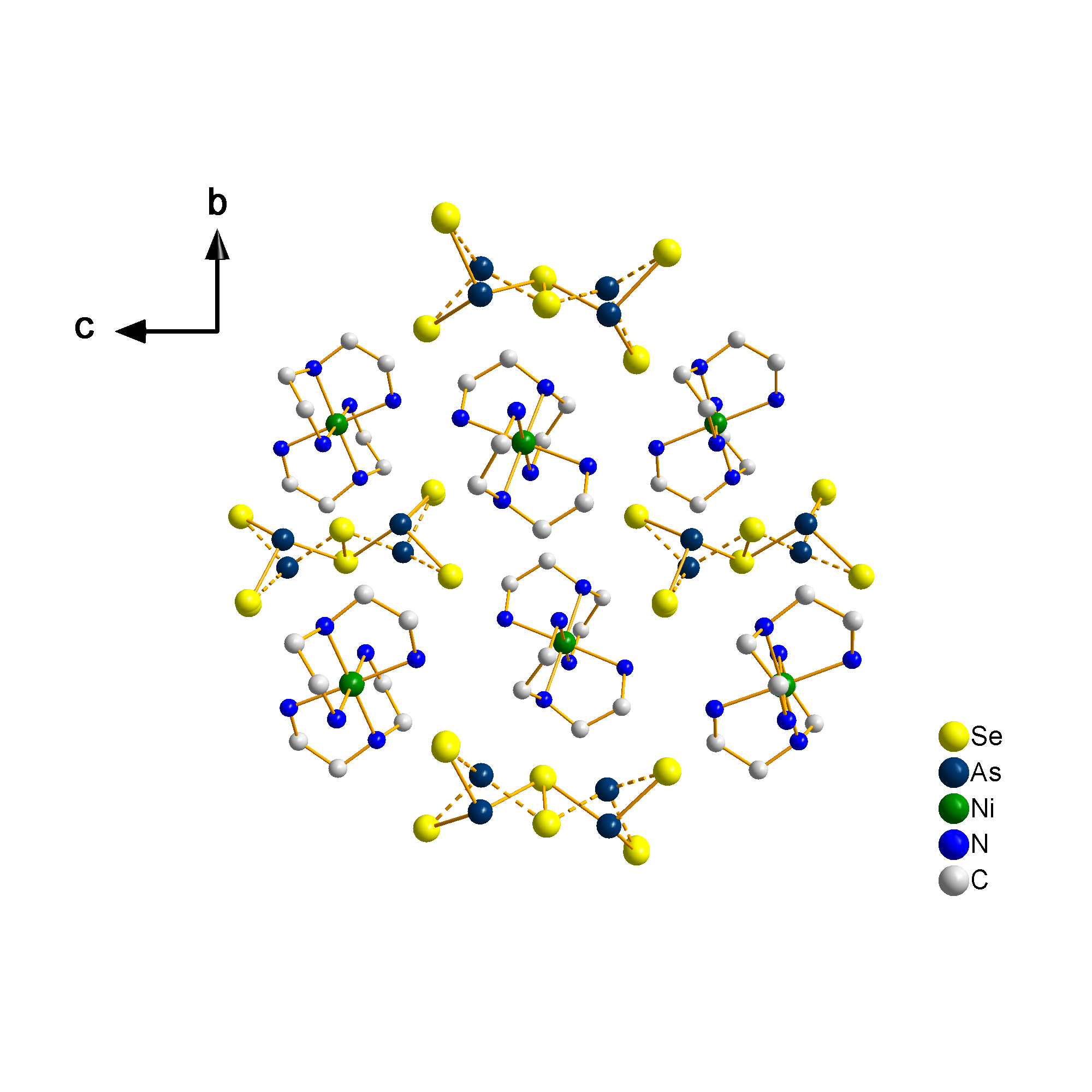


**Figure S1**. Crystal structure of compound **2** viewing the *a*-axis (hydrogen atoms are omitted for clarity).


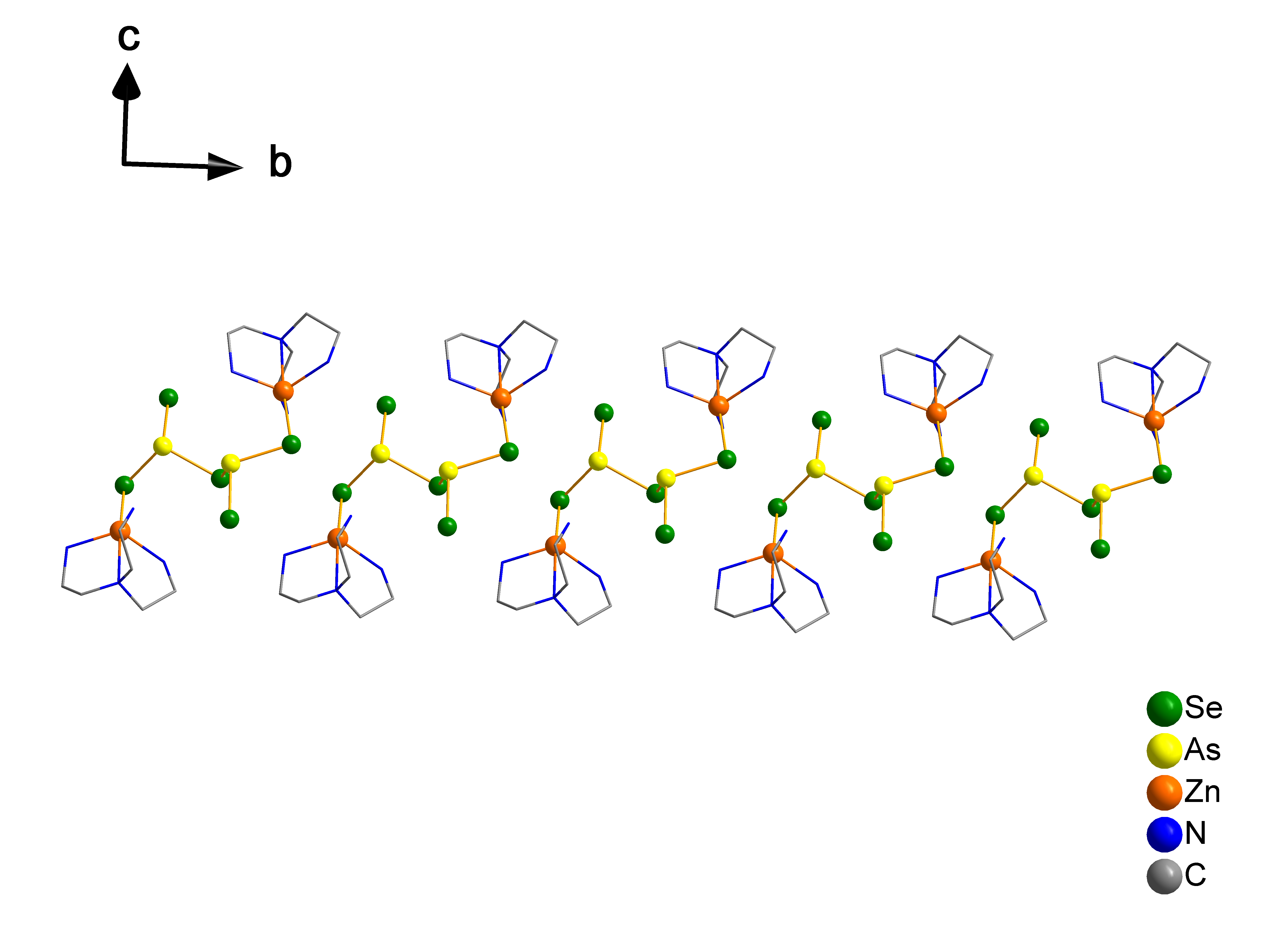


**Figure S2**. Crystal structure of compound **3** viewing the *a*-axis (hydrogen atoms are omitted for clarity).


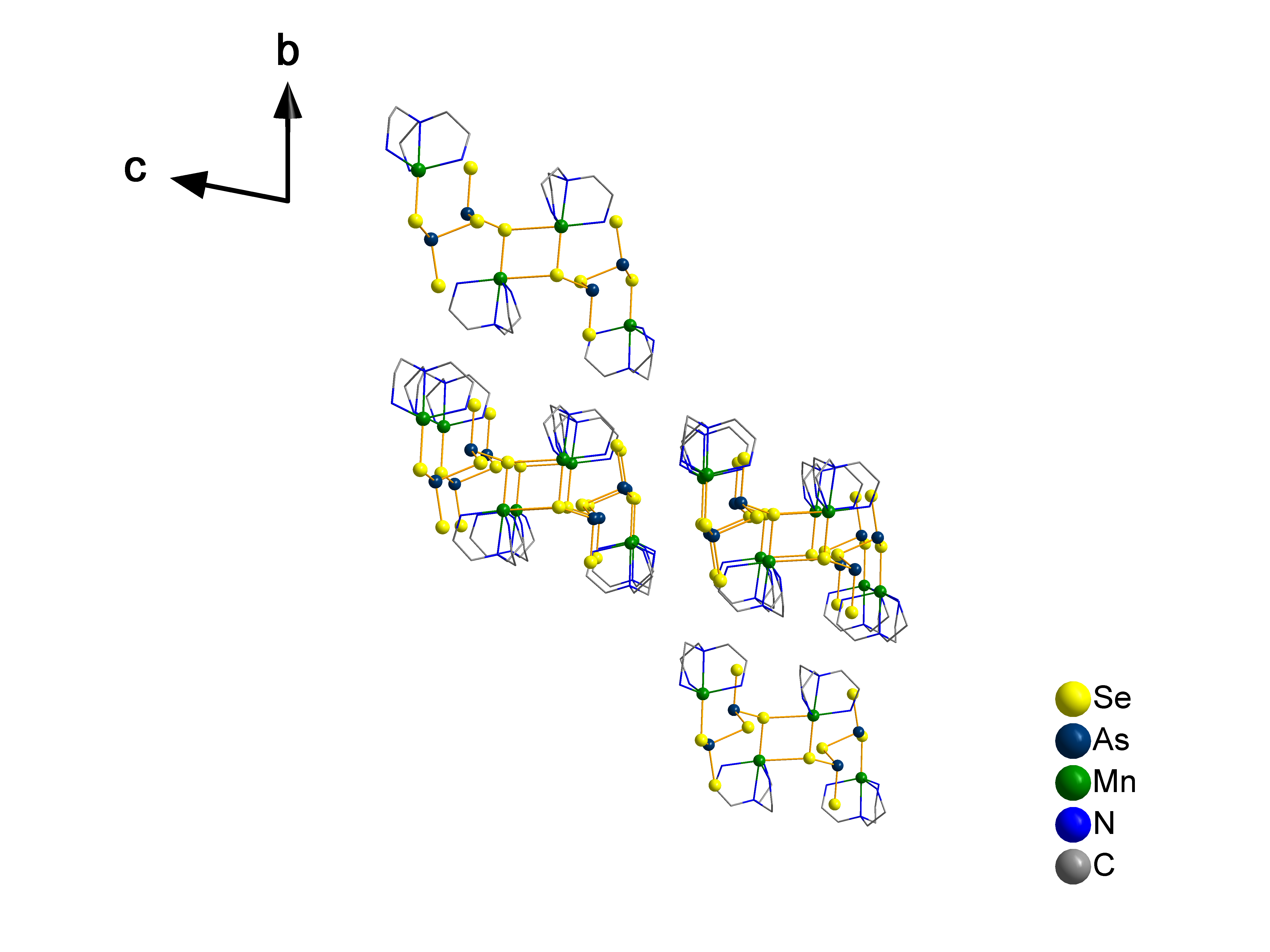


**Figure S3.** Crystal structure of compound **4** viewing the *a*-axis (hydrogen atoms are omitted for clarity).


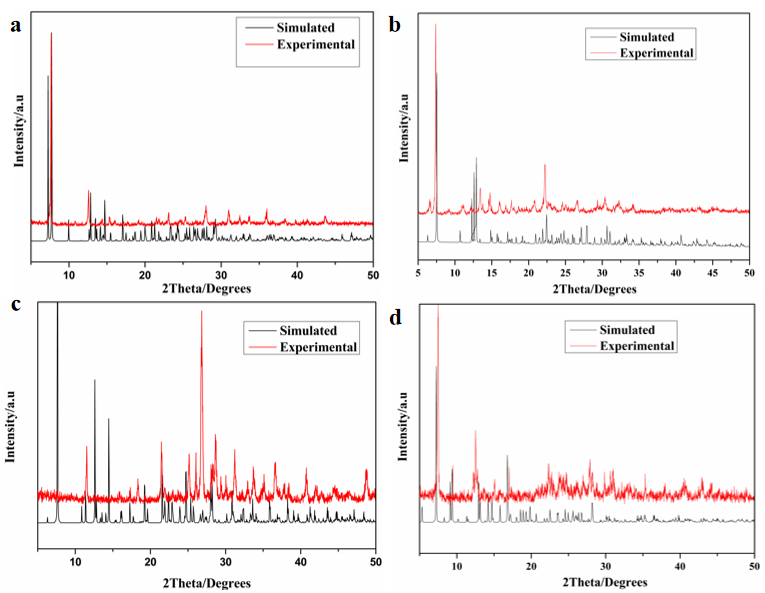


**Figure S4.** Powder X-ray Diffraction (PXRD) **1-4** (a-d).

**
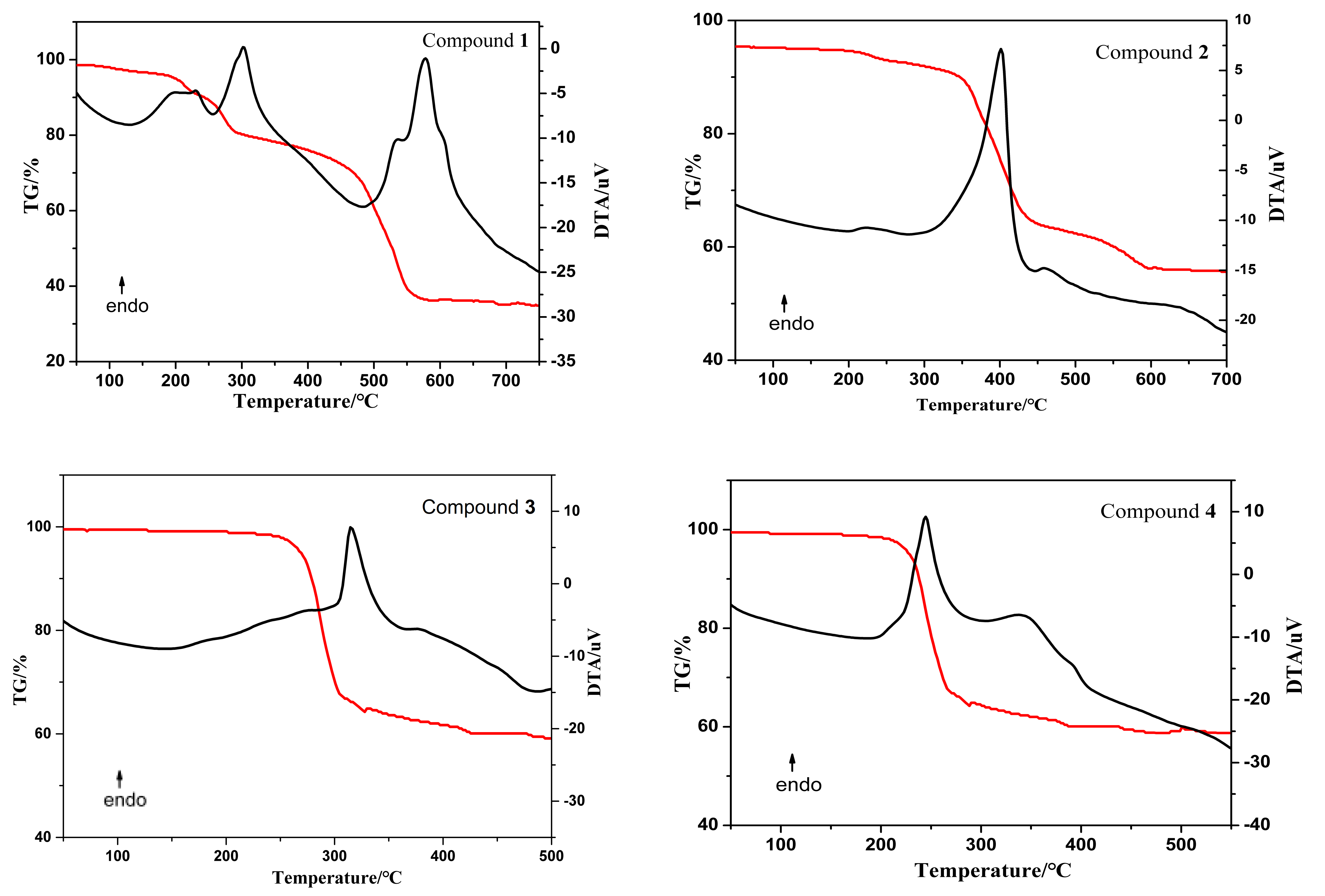
**

Figure S5. TG-DTA curves of the 1-4.


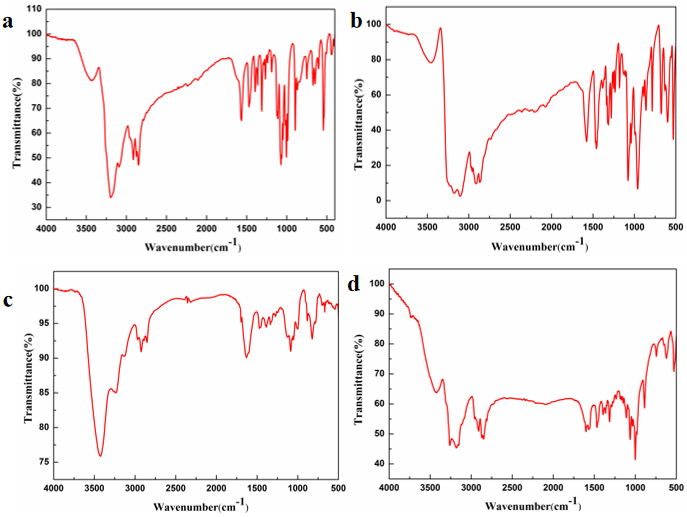


Figure S6. IR Spectra of the 1-4 (a-d).

**Table S1.** Summary of crystal data for compounds **1**-**4.**

| Parameter | **1** | **2** | **3** | **4** |
| --- | --- | --- | --- | --- |
| Identification code | [Co(tren)_2_H]AsSe_4_ | [Ni_2_(dien)_4_]As_2_Se_5_ | [Zn(tren)]_2_As_2_Se_5_ | [Mn(tren)]_2_As_2_Se_5_ |
| Empirical formula | C12H37AsCoN8Se4 | C16H52As2Ni2N12Se5 | C12H36As2Zn2N8Se5 | C12H36As2Mn2N8Se5 |
| Formula weight | 745.20 | 1072.38 | 967.87 | 947.01 |
| Crystal system | monoclinic | monoclinic | monoclinic | triclinic |
| Space group | *P*21/*n* | *P*21/*n* | *C*2/*c* | *P*‾1 |
| *a*/Å | 7.7063(2) | 9.4198(4) | 33.2294(6) | 8.2043(4) |
| *b*/Å | 12.9702(3) | 14.3828(5) | 8.09760(10) | 12.0848(7) |
| *c*/Å | 24.6488(7) | 12.6237(4) | 23.6865(4) | 14.3493(8) |
| *α*/^o^ | 90 | 90 | 90 | 78.421(5) |
| *β*/^o^ | 98.4320(10) | 91.500(3) | 122.3280(10) | 85.370(4) |
| *γ*/^o^ | 90 | 90 | 90 | 82.026(4) |
| Volume/Å^3^ | 2437.07(11) | 1709.71(11) | 5385.63(16) | 1378.21(13) |
| *Z* | 4 | 2 | 8 | 2 |
| *F*(000) | 1444 | 1046 | 3680 | 900 |
| *ρ*calc g/cm^3^ | 2.026 | 2.083 | 2.387 | 2.282 |
| Goodness-of-fit on *F*^2^ | 1.093 | 1.004 | 1.054 | 1.071 |
| Final *R* indexes  [*I*>=2σ (*I*)] | *R*1 = 0.0597,  *wR*2 = 0.1985 | *R*1 = 0.0552,  *wR*2 = 0.1266 | *R*1 = 0.0413,  *wR*2 = 0.1199 | *R*1 = 0.0718,  *wR*2 = 0.1990 |
| Final *R* indexes  [all data] | *R*1 = 0.0673,  *wR*2 = 0.2073 | *R*1 = 0.0847,  *wR*2 = 0.1456 | *R*1 = 0.0552,  *wR*2 = 0.1307 | *R*1 = 0.1100,  *wR*2 = 0.2320 |

**Table S2.** Selected bond lengths [Å] and angles [º] for **1.**

| Co1-N4 | 2.093(6) | C5-C6 | 1.522(11) | N3-C2-C1 | 110.1(7) |
| --- | --- | --- | --- | --- | --- |
| Co1-N5 | 2.057(8) | C7-C8 | 1.508(14) | N3-C4-C3 | 114.0(8) |
| Co1-N6 | 2.070(7) | C9-C10 | 1.497(13) | N3-C5-C6 | 112.9(7) |
| Co1-N7 | 2.258(6) | C11-C12 | 1.513(12) | N4-C6-C5 | 114.8(7) |
| Co1-N8 | 2.059(7) |  |  | N5-C7-C8 | 110.8(7) |
| Se1-As1 | 2.2679(13) | Se1-As1-Se2 | 108.60(5) | N6-C10-C9 | 110.1(7) |
| As1-Se2 | 2.2730(12) | Se1-As1-Se3 | 112.48(5) | N7-C8-C7 | 110.2(7) |
| As1-Se3 | 2.2825(11) | Se1-As1-Se4 | 109.12(5) | N7-C9-C10 | 110.9(7) |
| As1-Se4 | 2.2932(11) | Se2-As1-Se3 | 106.74(5) | N7-C11-C12 | 110.5(7) |
| N2-C3 | 1.434(12) | Se2-As1-Se4 | 108.43(5) | N8-C12-C11 | 110.0(7) |
| N3-C4 | 1.455(11) | Se3-As1-Se4 | 111.34(5) | C2-N3-C4 | 115.3(7) |
| N3-C5 | 1.458(10) | N4-Co1-N7 | 178.4(2) | C2-N3-C5 | 113.1(7) |
| N4-C6 | 1.446(11) | N5-Co1-N4 | 100.1(3) | C4-N3-C5 | 112.2(7) |
| N5-C7 | 1.445(13) | N5-Co1-N6 | 111.0(3) | C9-N7-C8 | 112.0(7) |
| N6-C10 | 1.462(12) | N5-Co1-N7 | 79.8(3) | C11-N7-C8 | 113.2(7) |
| N7-C8 | 1.484(11) | N5-Co1-N8 | 122.7(3) | C11-N7-C9 | 111.4(7) |
| N7-C9 | 1.473(11) | N6-Co1-N4 | 102.2(3) | C6-N4-Co1 | 116.7(5) |
| N7-C11 | 1.455(11) | N6-Co1-N7 | 79.3(3) | C7-N5-Co1 | 115.0(6) |
| N8-C12 | 1.449(11) | N8-Co1-N4 | 98.9(3) | C8-N7-Co1 | 105.7(5) |
| C1-N1 | 1.484(13) | N8-Co1-N6 | 116.8(3) | C9-N7-Co1 | 107.3(5) |
| C1-C2 | 1.513(13) | N8-Co1-N7 | 79.9(3) | C10-N6-Co1 | 114.0(5) |
| C2-N3 | 1.444(11) | N1-C1-C2 | 108.7(8) | C11-N7-Co1 | 106.7(5) |
| C3-C4 | 1.542(13) | N2-C3-C4 | 110.0(7) | C12-N8-Co1 | 113.2(5) |

**Table S3.** Selected bond lengths [Å] and angles [º] for **2.**

| As1B-Se3^1^ | 2.4404(19) | N4-Ni1 | 82.9(2) | Se3^1^-As1A-Se3 | 52.91(8) |
| --- | --- | --- | --- | --- | --- |
| As1B-Se3 | 1.8755(18) | N4-Ni1 | 82.2(2) | Se3-As1B-Se3 | 133.15(8) |
| Se1-As1B | 2.3248(14) | N4-Ni1 | 97.2(2) | Se3-As1B-Se2 | 118.48(8) |
| Se1-As1A | 2.3528(15) | N4-Ni1 | 97.7(2) | Se3-As1B-Se3^1^ | 52.98(8) |
| Se2-As1B | 2.3300(15) | N5-Ni1 | 178.8(2) | Se3-As1B-Se3 | 133.15(8) |
| Se2-As1A | 2.2843(17) | N3-Ni1 | 89.8(2) | Se3-As1B-Se2 | 118.48(8) |
| Se3-Se3^1^ | 1.990(3) | N3-Ni1 | 179.3(2) | Se3-As1B-Se3^1^ | 52.98(8) |
| Se3-As1A^1^ | 1.881(2) | N3-Ni1 | 89.2(2) | Se3^1^-Se3-As1B^1^ | 48.80(7) |
| Se3-As1A | 2.442(2) | N6-Ni1 | 89.9(2) | Se3^1^-Se3-As1A | 48.93(7) |
| Ni1-N4 | 2.101(6) | N6-Ni1 | 91.1(2) | C6-N4-Ni1 | 107.5(5) |
| Ni1-N5 | 2.127(6) | N1-Ni1 | 178.9(2) | C7-N4-Ni1 | 107.5(4) |
| Ni1-N3 | 2.111(6) | N1-Ni1 | 96.3(2) | C7-N4-C6 | 116.8(6) |
| Ni1-N6 | 2.108(6) | N1-Ni1 | 97.1(2) | C5-N5-Ni1 | 109.0(4) |
| Ni1-N2 | 2.135(6) | N1-Ni1 | 83.5(3) | C8-N3-Ni1 | 111.8(4) |
| Ni1-N1 | 2.081(6) | N1-Ni1 | 83.2(2) | C4-N6-Ni1 | 109.7(5) |
| N4-C6 | 1.475(9) | As1A^1^-Se3-As1B^1^ | 30.60(6) | C1-N2-Ni1 | 109.0(5) |
| N4-C7 | 1.453(9) | As1A^1^-Se3-Se3^1^ | 78.17(9) | C2-N1-Ni1 | 109.2(5) |
| N5-C5 | 1.458(9) | As1A^1^-Se3-As1A | 127.09(8) | C2-N1-C3 | 116.3(6) |
| N3-C8 | 1.473(10) | As1B-Se3-As1A^1^ | 154.46(11) | C3-N1-Ni1 | 106.7(5) |
| N6-C4 | 1.464(11) | Se1-As1A-Se3 | 107.14(7) | N5-C5-C6 | 110.1(6) |
| N2-C1 | 1.474(10) | Se1-As1B-Se2 | 104.82(5) | N2-C1-C2 | 110.5(6) |
| N1-C2 | 1.475(10) | Se1-As1B-Se3^1^ | 99.69(6) | N4-C6-C5 | 112.3(6) |
| N1-C3 | 1.494(11) | Se2-As1A-Se1 | 105.38(6) | N4-C7-C8 | 113.0(6) |
| C5-C6 | 1.499(11) | Se2-As1A-Se3 | 99.97(7) | N3-C8-C7 | 109.7(6) |
| C1-C2 | 1.517(12) | Se2-As1B-Se3^1^ | 106.58(7) | N1-C2-C1 | 112.0(6) |
| C7-C8 | 1.520(11) | Se3^1^-As1A-Se1 | 118.30(8) | N6-C4-C3 | 110.9(6) |
| C4-C3 | 1.505(13) | Se3^1^-As1A-Se2 | 133.11(9) | N1-C3-C4 | 110.7(7) |

Symmetry transformations used to generate equivalent atoms: ^#1^1-X, 1-Y, 2-Z for **2**.

**Table S4.** Selected bond lengths [Å] and angles [º] for **3.**

| Se1-As2 | 2.3349(11) | C9-C10 | 1.530(9) | N8-Zn2-N6 | 118.1(3) |
| --- | --- | --- | --- | --- | --- |
| Se1-Zn1 | 2.4409(11) | C11-C12 | 1.520(10) | C1-N1-Zn1 | 105.8(4) |
| Se2-As2 | 2.3181(10) |  |  | C1-N1-C3 | 113.3(5) |
| Se3-As1 | 2.4528(10) | As2-Se1-Zn1 | 97.04(4) | C1-N1-C5 | 112.6(5) |
| Se3-As2 | 2.4685(10) | As1-Se3-As2 | 86.92(3) | C3-N1-Zn1 | 104.7(4) |
| Se4-As1 | 2.3232(10) | As1-Se5-Zn2 | 96.13(4) | C3-N1-C5 | 113.8(5) |
| Se5-As1 | 2.3633(10) | Se4-As1-Se3 | 103.05(4) | C5-N1-Zn1 | 105.5(4) |
| Se5-Zn2 | 2.4750(11) | Se4-As1-Se5 | 105.74(4) | C2-N2-Zn1 | 112.2(4) |
| Zn1-N1 | 2.416(6) | Se5-As1-Se3 | 104.05(4) | C4-N3-Zn1 | 112.3(4) |
| Zn1-N2 | 2.075(6) | Se1-As2-Se3 | 100.92(4) | C6-N4-Zn1 | 115.5(4) |
| Zn1-N3 | 2.078(6) | Se2-As2-Se1 | 103.48(4) | C7-N5-Zn2 | 106.2(4) |
| Zn1-N4 | 2.096(6) | Se2-As2-Se3 | 104.13(4) | C9-N5-Zn2 | 105.0(4) |
| Zn2-N5 | 2.345(6) | N1-Zn1-Se1 | 171.07(14) | C9-N5-C7 | 113.5(5) |
| Zn2-N6 | 2.110(6) | N2-Zn1-Se1 | 102.69(16) | C9-N5-C11 | 113.6(6) |
| Zn2-N7 | 2.074(6) | N2-Zn1-N1 | 77.5(2) | C11-N5-Zn2 | 106.0(4) |
| Zn2-N8 | 2.082(6) | N2-Zn1-N3 | 113.7(2) | C11-N5-C7 | 111.7(5) |
| N1-C1 | 1.456(8) | N2-Zn1-N4 | 123.8(2) | C8-N6-Zn2 | 113.6(4) |
| N1-C3 | 1.474(8) | N3-Zn1-Se1 | 110.03(15) | C10-N7-Zn2 | 114.1(4) |
| N1-C5 | 1.475(8) | N3-Zn1-N1 | 77.6(2) | C12-N8-Zn2 | 112.7(4) |
| N2-C2 | 1.489(9) | N3-Zn1-N4 | 108.3(2) | N1-C1-C2 | 111.0(6) |
| N3-C4 | 1.462(9) | N4-Zn1-Se1 | 95.98(16) | N2-C2-C1 | 109.0(6) |
| N4-C6 | 1.475(9) | N4-Zn1-N1 | 76.8(2) | N1-C3-C4 | 109.9(5) |
| N5-C7 | 1.477(8) | N5-Zn2-Se5 | 174.84(14) | N3-C4-C3 | 108.4(5) |
| N5-C9 | 1.456(9) | N6-Zn2-Se5 | 97.15(18) | N1-C5-C6 | 109.9(5) |
| N5-C11 | 1.467(9) | N6-Zn2-N5 | 78.1(2) | N4-C6-C5 | 110.1(5) |
| N6-C8 | 1.462(9) | N7-Zn2-Se5 | 102.06(16) | N5-C7-C8 | 110.7(6) |
| N7-C10 | 1.458(9) | N7-Zn2-N5 | 78.5(2) | N6-C8-C7 | 110.1(6) |
| N8-C12 | 1.458(9) | N7-Zn2-N6 | 116.9(3) | N5-C9-C10 | 110.9(5) |
| C1-C2 | 1.521(10) | N7-Zn2-N8 | 113.1(2) | N7-C10-C9 | 108.1(6) |
| C3-C4 | 1.531(10) | N8-Zn2-Se5 | 105.85(16) | N5-C11-C12 | 110.4(6) |
| C5-C6 | 1.516(10) | N8-Zn2-N5 | 78.4(2) | N8-C12-C11 | 109.3(6) |
| C7-C8 | 1.512(10) |  |  |  |  |

**Table S5.** Selected bond lengths [Å] and angles [º] for **4.**

| Se1-As2 | 2.3664(16) | As2-Se1-Mn2 | 94.95(6) | N7-Mn2-Se1 | 176.1(2) |
| --- | --- | --- | --- | --- | --- |
| Se1-Mn2 | 2.551(2) | As2-Se3-As1 | 87.79(5) | N8-Mn2-Se1 | 99.6(3) |
| Se2-As2 | 2.3291(16) | As1-Se5-Mn1 | 101.93(7) | N8-Mn2-N7 | 76.9(3) |
| Se3-As1 | 2.4628(17) | As1-Se5-Mn1^1^ | 136.69(8) | C1-N1-Mn1 | 108.2(7) |
| Se3-As2 | 2.4520(16) | Mn1-Se5-Mn1^1^ | 98.49(7) | C2-N2-Mn1 | 107.5(7) |
| Se4-As1 | 2.3291(17) | Se4-As1-Se3 | 103.93(6) | C2-N2-C3 | 107.3(12) |
| Se5-As1 | 2.2952(18) | Se5-As1-Se3 | 102.15(7) | C3-N2-Mn1 | 102.2(7) |
| Se5-Mn1 | 2.525(2) | Se5-As1-Se4 | 103.37(6) | C5-N2-Mn1 | 108.1(8) |
| Se5-Mn1^1^ | 2.932(2) | Se1-As2-Se3 | 102.04(6) | C5-N2-C2 | 115.6(13) |
| Mn1-N1 | 2.225(9) | Se2-As2-Se1 | 105.29(6) | C5-N2-C3 | 115.1(12) |
| Mn1-N2 | 2.368(9) | Se2-As2-Se3 | 104.07(6) | C4-N3-Mn1 | 112.4(7) |
| Mn1-N3 | 2.262(9) | Se5-Mn1-Se5^1^ | 81.50(7) | C6-N4-Mn1 | 112.1(8) |
| Mn1-N4 | 2.239(10) | N1-Mn1-Se5_1_ | 84.4(3) | C7-N5-Mn2 | 112.9(7) |
| Mn2-N5 | 2.192(10) | N1-Mn1-Se5 | 100.8(3) | C10-N6-Mn2 | 111.5(7) |
| Mn2-N6 | 2.200(9) | N1-Mn1-N2 | 76.5(3) | C8-N7-Mn2 | 106.4(6) |
| Mn2-N7 | 2.363(9) | N1-Mn1-N3 | 142.2(4) | C9-N7-Mn2 | 107.3(6) |
| Mn2-N8 | 2.231(10) | N1-Mn1-N4 | 104.0(4) | C9-N7-C8 | 112.3(9) |
| N1-C1 | 1.457(18) | N2-Mn1-Se5^1^ | 101.2(2) | C9-N7-C11 | 111.4(9) |
| N2-C2 | 1.452(15) | N2-Mn1-Se5 | 175.9(2) | C11-N7-Mn2 | 108.0(7) |
| N2-C3 | 1.490(16) | N3-Mn1-Se5 | 107.8(3) | C11-N7-C8 | 111.1(9) |
| N2-C5 | 1.416(17) | N3-Mn1-Se5^1^ | 76.2(2) | C12-N8-Mn2 | 111.2(7) |
| N3-C4 | 1.437(15) | N3-Mn1-N2 | 76.0(3) | C2-C1-N1 | 113.2(12) |
| N4-C6 | 1.477(17) | N4-Mn1-Se51 | 169.9(3) | C1-C2-N2 | 117.1(11) |
| N5-C7 | 1.447(15) | N4-Mn1-Se5 | 102.0(3) | N2-C3-C4 | 111.9(11) |
| N6-C10 | 1.487(15) | N4-Mn1-N2 | 75.8(4) | N3-C4-C3 | 115.5(10) |
| N7-C8 | 1.467(14) | N4-Mn1-N3 | 93.7(4) | N2-C5-C6 | 115.2(12) |
| N7-C9 | 1.458(14) | N5-Mn2-Se1 | 103.8(3) | N4-C6-C5 | 108.9(11) |
| N7-C11 | 1.465(13) | N5-Mn2-N6 | 112.0(4) | N5-C7-C8 | 109.0(9) |
| N8-C12 | 1.485(17) | N5-Mn2-N7 | 76.4(3) | N7-C8-C7 | 111.5(10) |
| C1-C2 | 1.445(19) | N5-Mn2-N8 | 114.7(4) | N7-C9-C10 | 112.4(9) |
| C3-C4 | 1.494(18) | N6-Mn2-Se1 | 106.6(3) | N6-C10-C9 | 108.1(9) |
| C5-C6 | 1.50(2) | N6-Mn2-N7 | 76.8(3) | N7-C11-C12 | 112.2(10) |
| C7-C8 | 1.504(16) | N6-Mn2-N8 | 117.8(4) | N8-C12-C11 | 111.5(10) |
| C9-C10 | 1.510(17) |  |  |  |  |
| C11-C12 | 1.496(17) |  |  |  |  |

Symmetry transformations used to generate equivalent atoms: ^#1^2-X, 1-Y, 2-Z for **4**.

1. * Corresponding author.

   E-mail address: baiymh@imnu.edu.cn. [↑](#footnote-ref-1)
