# Supplementary material for: Strong Photocurrent Response of Selenoarsenates With Different Transition Metal Complexes as Structure-Directing Agents
Source: Front Chem. 2022 May 5;10:890496. doi: 10.3389/fchem.2022.890496 (PMC9117718; doi:10.3389/fchem.2022.890496)
Supplement: Supplementary file 4 [file DataSheet5.docx]

**Highlights**

**Strong Photocurrent Response of Selenoarsenates with Different Transition Metal Complexes as Structure-Directing Agents**

Xinyu Tian^1^, Gele Teri^1^, Muge Shele^1^, Namila E^1^, Liming Qi^1^, Min Liu^1^ and Menghe Baiyin^1^^[[1]](#footnote-1)^*

(College of Chemistry & Environmental Science, Inner Mongolia University Key Laboratory of Advanced Materials Chemistry and Devices (AMC&DLab), Inner Mongolia Normal University, Hohhot, Inner Mongolia 010022, P.R. China)

- Under solvothermal conditions, we synthesized four novel selenoarsenates utilizing transition-metal complexes (TMC) as structure-directing agents.
- In compounds **3** and **4**, transition metal complexes are linked to dimers of [As_2_Se_5_]^4-^.
- They exhibit higher transient photocurrent responses than other chalcogenides.

1. * Corresponding author.

   E-mail address: baiymh@imnu.edu.cn. [↑](#footnote-ref-1)
