# Supplementary material for: Strong Photocurrent Response of Selenoarsenates With Different Transition Metal Complexes as Structure-Directing Agents
Source: Front Chem. 2022 May 5;10:890496. doi: 10.3389/fchem.2022.890496 (PMC9117718; doi:10.3389/fchem.2022.890496)

checkCIF/PLATON report

Structure factors have been supplied for datablock(s) s

THIS REPORT IS FOR GUIDANCE ONLY. IF USED AS PART OF A REVIEW PROCEDURE FOR PUBLICATION, IT SHOULD NOT REPLACE THE EXPERTISE OF AN EXPERIENCED CRYSTALLOGRAPHIC REFEREE.

No syntax errors found. CIF dictionary Interpreting this report

Datablock: s

```
Bond precision:  C-C = 0.0115 A          Wavelength=0.71073

Cell:           a=33.2294(6)    b=8.0976(1)        c=23.6865(4)
                alpha=90        beta=122.328(1)    gamma=90
Temperature:    150 K

                Calculated                 Reported
Volume          5385.63(16)                5385.63(16)
Space group     C 2/c                      C 1 2/c 1
Hall group      -C 2yc                     -C 2yc
Moiety formula  C12 H36 As2 N8 Se5 Zn2     C12 H36 As2 N8 Se5 Zn2
Sum formula     C12 H36 As2 N8 Se5 Zn2     C12 H36 As2 N8 Se5 Zn2
Mr              967.91                     967.87
Dx,g cm-3       2.388                      2.387
Z               8                          8
Mu (mm-1)       10.996                     10.996
F000            3680.0                     3680.0
F000'           3683.76
h,k,lmax        41,10,29                   41,10,29
Nref            5504                       5502
Tmin,Tmax                                  0.007,0.028
Tmin'

Correction method= # Reported T Limits: Tmin=0.007 Tmax=0.028
AbsCorr = MULTI-SCAN

Data completeness= 1.000          Theta(max)= 26.383

R(reflections)= 0.0413( 4434)     wR2(reflections)= 0.1307( 5502)

S = 1.054                  Npar= 262
```

The following ALERTS were generated. Each ALERT has the format
test-name_ALERT_alert-type_alert-level.
Click on the hyperlinks for more details of the test.

Alert level B

```
PLAT230_ALERT_2_B Hirshfeld Test Diff for    Se1      --As2      .       23.4 s.u.
PLAT230_ALERT_2_B Hirshfeld Test Diff for    Se5      --As1      .       19.0 s.u.
PLAT232_ALERT_2_B Hirshfeld Test Diff (M-X)  Se1      --Zn1      .       19.3 s.u.
```

Alert level C

```
PLAT053_ALERT_1_C Minimum Crystal Dimension Missing (or Error) ...     Please Check
PLAT054_ALERT_1_C Medium  Crystal Dimension Missing (or Error) ...     Please Check
PLAT055_ALERT_1_C Maximum Crystal Dimension Missing (or Error) ...     Please Check
PLAT230_ALERT_2_C Hirshfeld Test Diff for    Se3      --As1      .        6.0 s.u.
PLAT341_ALERT_3_C Low Bond Precision on  C-C Bonds ...............     0.0115 Ang.
PLAT420_ALERT_2_C D-H Bond Without Acceptor  N4       --H4A      .     Please Check
PLAT420_ALERT_2_C D-H Bond Without Acceptor  N6       --H6A      .     Please Check
PLAT420_ALERT_2_C D-H Bond Without Acceptor  N6       --H6B      .     Please Check
PLAT972_ALERT_2_C Check Calcd Resid. Dens.  0.40A   From Se1          -2.26 eA-3
PLAT972_ALERT_2_C Check Calcd Resid. Dens.  0.38A   From Se1          -2.16 eA-3
PLAT973_ALERT_2_C Check Calcd Positive Resid. Density on       Zn2       1.07 eA-3
PLAT976_ALERT_2_C Check Calcd Resid. Dens.  1.06A   From N8           -0.45 eA-3
PLAT977_ALERT_2_C Check Negative Difference Density on H6B            -0.32 eA-3
```

Alert level G

```
PLAT007_ALERT_5_G Number of Unrefined Donor-H Atoms ..............        12 Report
PLAT083_ALERT_2_G SHELXL Second Parameter in WGHT  Unusually Large      55.11 Why ?
PLAT128_ALERT_4_G Alternate Setting for Input Space Group     C2/c       I2/a Note
PLAT232_ALERT_2_G Hirshfeld Test Diff (M-X)  Se5      --Zn2      .        9.5 s.u.
PLAT794_ALERT_5_G Tentative Bond Valency for Zn1       (II)      .       1.83 Info
PLAT794_ALERT_5_G Tentative Bond Valency for Zn2       (II)      .       1.79 Info
PLAT910_ALERT_3_G Missing # of FCF Reflection(s) Below Theta(Min).          2 Note
PLAT912_ALERT_4_G Missing # of FCF Reflections Above STh/L=  0.600          1 Note
PLAT978_ALERT_2_G Number C-C Bonds with Positive Residual Density.          1 Info
```

```
   0 ALERT level A = Most likely a serious problem - resolve or explain
   3 ALERT level B = A potentially serious problem, consider carefully
  13 ALERT level C = Check. Ensure it is not caused by an omission or oversight
   9 ALERT level G = General information/check it is not something unexpected

   3 ALERT type 1 CIF construction/syntax error, inconsistent or missing data
  15 ALERT type 2 Indicator that the structure model may be wrong or deficient
   2 ALERT type 3 Indicator that the structure quality may be low
   2 ALERT type 4 Improvement, methodology, query or suggestion
   3 ALERT type 5 Informative message, check
```

It is advisable to attempt to resolve as many as possible of the alerts in all categories. Often the minor alerts point to easily fixed oversights, errors and omissions in your CIF or refinement strategy, so attention to these fine details can be worthwhile. In order to resolve some of the more serious problems it may be necessary to carry out additional measurements or structure refinements. However, the purpose of your study may justify the reported deviations and the more serious of these should normally be commented upon in the discussion or experimental section of a paper or in the "special_details" fields of the CIF. checkCIF was carefully designed to identify outliers and unusual parameters, but every test has its limitations and alerts that are not important in a particular case may appear. Conversely, the absence of alerts does not guarantee there are no aspects of the results needing attention. It is up to the individual to critically assess their own results and, if necessary, seek expert advice.

Publication of your CIF in IUCr journals

A basic structural check has been run on your CIF. These basic checks will be run on all CIFs submitted for publication in IUCr journals (*Acta Crystallographica*, *Journal of Applied Crystallography*, *Journal of Synchrotron Radiation*); however, if you intend to submit to *Acta Crystallographica Section C* or *E* or *IUCrData*, you should make sure that full publication checks are run on the final version of your CIF prior to submission.

Publication of your CIF in other journals

Please refer to the *Notes for Authors* of the relevant journal for any special instructions relating to CIF submission.

PLATON version of 13/07/2021; check.def file version of 13/07/2021

Datablock s - ellipsoid plot

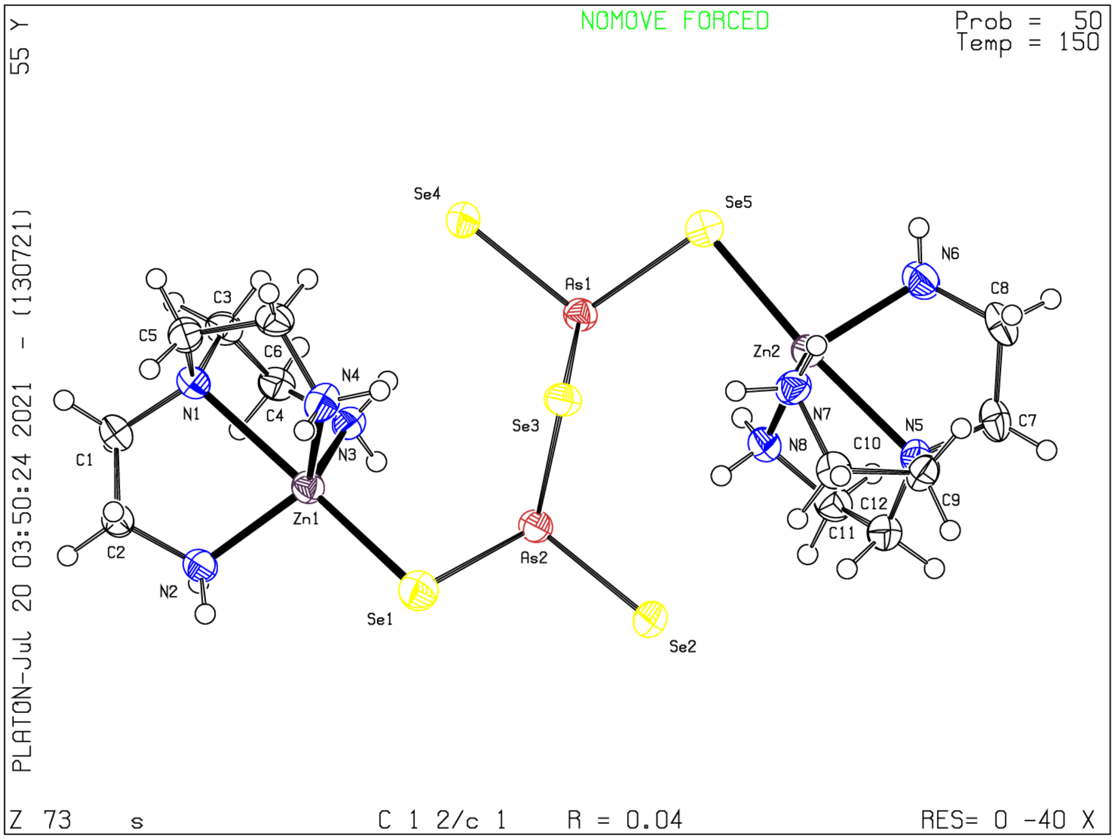

Supplement: Supplementary file 5 [file DataSheet3.PDF]
