# Supplementary material for: Strong Photocurrent Response of Selenoarsenates With Different Transition Metal Complexes as Structure-Directing Agents
Source: Front Chem. 2022 May 5;10:890496. doi: 10.3389/fchem.2022.890496 (PMC9117718; doi:10.3389/fchem.2022.890496)

# checkCIF/PLATON report

You have not supplied any structure factors. As a result the full set of tests cannot be run.

THIS REPORT IS FOR GUIDANCE ONLY. IF USED AS PART OF A REVIEW PROCEDURE FOR PUBLICATION, IT SHOULD NOT REPLACE THE EXPERTISE OF AN EXPERIENCED CRYSTALLOGRAPHIC REFEREE.

No syntax errors found. CIF dictionary Interpreting this report

## Datablock: s

---

```
Bond precision:   C-C = 0.0127 A          Wavelength=0.71073

Cell:              a=7.7063(2)     b=12.9702(3)        c=24.6488(7)
                   alpha=90        beta=98.432(1)      gamma=90
Temperature:       150 K
```

| | Calculated | Reported |
|---|---|---|
| Volume | 2437.07(11) | 2437.07(11) |
| Space group | P 21/n | P 1 21/n 1 |
| Hall group | -P 2yn | -P 2yn |
| Moiety formula | C12 H37 Co N8, As Se4 | As Se4, C12 H37 Co N8 |
| Sum formula | C12 H37 As Co N8 Se4 | C12 H39 As Co N8 Se4 |
| Mr | 743.19 | 745.20 |
| Dx,g cm-3 | 2.026 | 2.031 |
| Z | 4 | 4 |
| Mu (mm-1) | 8.040 | 8.041 |
| F000 | 1444.0 | 1452.0 |
| F000' | 1444.74 | |
| h,k,lmax | 9,16,30 | 9,16,30 |
| Nref | 4980 | 4978 |
| Tmin,Tmax | | 0.008,0.028 |
| Tmin' | | |

```
Correction method= # Reported T Limits: Tmin=0.008 Tmax=0.028
AbsCorr = MULTI-SCAN

Data completeness= 1.000           Theta(max)= 26.372

R(reflections)= 0.0606( 4298)                       wR2(reflections)=
                                                    0.2268( 4978)
S = 1.834                   Npar= 237
```

The following ALERTS were generated. Each ALERT has the format
**test-name_ALERT_alert-type_alert-level.**
Click on the hyperlinks for more details of the test.

---

## Alert level B

```
PLAT416_ALERT_2_B Short Intra D-H..H-D       H1        ..H2AD     .       1.68 Ang.
                                                       x,y,z  =      1_555 Check
PLAT416_ALERT_2_B Short Intra D-H..H-D       H1AE      ..H2AD     .       1.35 Ang.
                                                       x,y,z  =      1_555 Check
```

---

## Alert level C

```
DIFMN02_ALERT_2_C  The minimum difference density is < -0.1*ZMAX*0.75
            _refine_diff_density_min given =     -3.063
            Test value =     -2.550
DIFMN03_ALERT_1_C  The minimum difference density is < -0.1*ZMAX*0.75
            The relevant atom site should be identified.
DIFMX02_ALERT_1_C  The maximum difference density is > 0.1*ZMAX*0.75
            The relevant atom site should be identified.
PLAT041_ALERT_1_C Calc. and Reported SumFormula    Strings  Differ     Please Check
PLAT043_ALERT_1_C Calculated and Reported Mol. Weight Differ by ..       2.01 Check
PLAT053_ALERT_1_C Minimum Crystal Dimension Missing (or Error) ...     Please Check
PLAT054_ALERT_1_C Medium  Crystal Dimension Missing (or Error) ...     Please Check
PLAT055_ALERT_1_C Maximum Crystal Dimension Missing (or Error) ...     Please Check
PLAT068_ALERT_1_C Reported F000 Differs from Calcd (or Missing)...     Please Check
PLAT097_ALERT_2_C Large Reported Max.  (Positive) Residual Density       3.40 eA-3
PLAT098_ALERT_2_C Large Reported Min.  (Negative) Residual Density      -3.06 eA-3
PLAT244_ALERT_4_C Low    'Solvent' Ueq as Compared to Neighbors of        As1 Check
PLAT341_ALERT_3_C Low Bond Precision on  C-C Bonds ..............    0.01267 Ang.
PLAT416_ALERT_2_C Short Intra D-H..H-D       H1        ..H1AE     .       1.97 Ang.
                                                       x,y,z  =      1_555 Check
PLAT601_ALERT_2_C Unit Cell Contains Solvent Accessible VOIDS of .         39 Ang**3
```

---

## Alert level G

```
FORMU01_ALERT_1_G  There is a discrepancy between the atom counts in the
            _chemical_formula_sum and _chemical_formula_moiety. This is
            usually due to the moiety formula being in the wrong format.
            Atom count from _chemical_formula_sum:   C12 H39 As1 Co1 N8 Se4
            Atom count from _chemical_formula_moiety:C12 H37 As1 Co1 N8 Se4
FORMU01_ALERT_2_G  There is a discrepancy between the atom counts in the
            _chemical_formula_sum and the formula from the _atom_site* data.
            Atom count from _chemical_formula_sum:C12 H39 As1 Co1 N8 Se4
            Atom count from the _atom_site data:  C12 H37 As1 Co1 N8 Se4
CELLZ01_ALERT_1_G Difference between formula and atom_site contents detected.
CELLZ01_ALERT_1_G WARNING: H atoms missing from atom site list. Is this intentional?
           From the CIF: _cell_formula_units_Z    4
           From the CIF: _chemical_formula_sum  C12 H39 As Co N8 Se4
           TEST: Compare cell contents of formula and atom_site data

           atom    Z*formula  cif sites diff
           C         48.00     48.00    0.00
           H        156.00    148.00    8.00
           As         4.00      4.00    0.00
```

```
          Co        4.00      4.00    0.00
          N        32.00     32.00    0.00
          Se       16.00     16.00    0.00
PLAT007_ALERT_5_G Number of Unrefined Donor-H Atoms .............         13 Report
PLAT012_ALERT_1_G No       _shelx_res_checksum Found in CIF ......     Please Check
PLAT042_ALERT_1_G Calc. and Reported Moiety Formula Strings Differ     Please Check
PLAT720_ALERT_4_G Number of Unusual/Non-Standard Labels ..........         20 Note
PLAT794_ALERT_5_G Tentative Bond Valency for Co3       (II)      .       1.78 Info
```

---

```
   0 ALERT level A = Most likely a serious problem - resolve or explain
   2 ALERT level B = A potentially serious problem, consider carefully
  15 ALERT level C = Check. Ensure it is not caused by an omission or oversight
   9 ALERT level G = General information/check it is not something unexpected

  13 ALERT type 1 CIF construction/syntax error, inconsistent or missing data
   8 ALERT type 2 Indicator that the structure model may be wrong or deficient
   1 ALERT type 3 Indicator that the structure quality may be low
   2 ALERT type 4 Improvement, methodology, query or suggestion
   2 ALERT type 5 Informative message, check
```

---

It is advisable to attempt to resolve as many as possible of the alerts in all categories. Often the minor alerts point to easily fixed oversights, errors and omissions in your CIF or refinement strategy, so attention to these fine details can be worthwhile. In order to resolve some of the more serious problems it may be necessary to carry out additional measurements or structure refinements. However, the purpose of your study may justify the reported deviations and the more serious of these should normally be commented upon in the discussion or experimental section of a paper or in the "special_details" fields of the CIF. checkCIF was carefully designed to identify outliers and unusual parameters, but every test has its limitations and alerts that are not important in a particular case may appear. Conversely, the absence of alerts does not guarantee there are no aspects of the results needing attention. It is up to the individual to critically assess their own results and, if necessary, seek expert advice.

**Publication of your CIF in IUCr journals**

A basic structural check has been run on your CIF. These basic checks will be run on all CIFs submitted for publication in IUCr journals (*Acta Crystallographica*, *Journal of Applied Crystallography*, *Journal of Synchrotron Radiation*); however, if you intend to submit to *Acta Crystallographica Section C* or *E* or *IUCrData*, you should make sure that full publication checks are run on the final version of your CIF prior to submission.

**Publication of your CIF in other journals**

Please refer to the *Notes for Authors* of the relevant journal for any special instructions relating to CIF submission.

---

**PLATON version of 13/07/2021; check.def file version of 13/07/2021**

**Datablock s** - ellipsoid plot

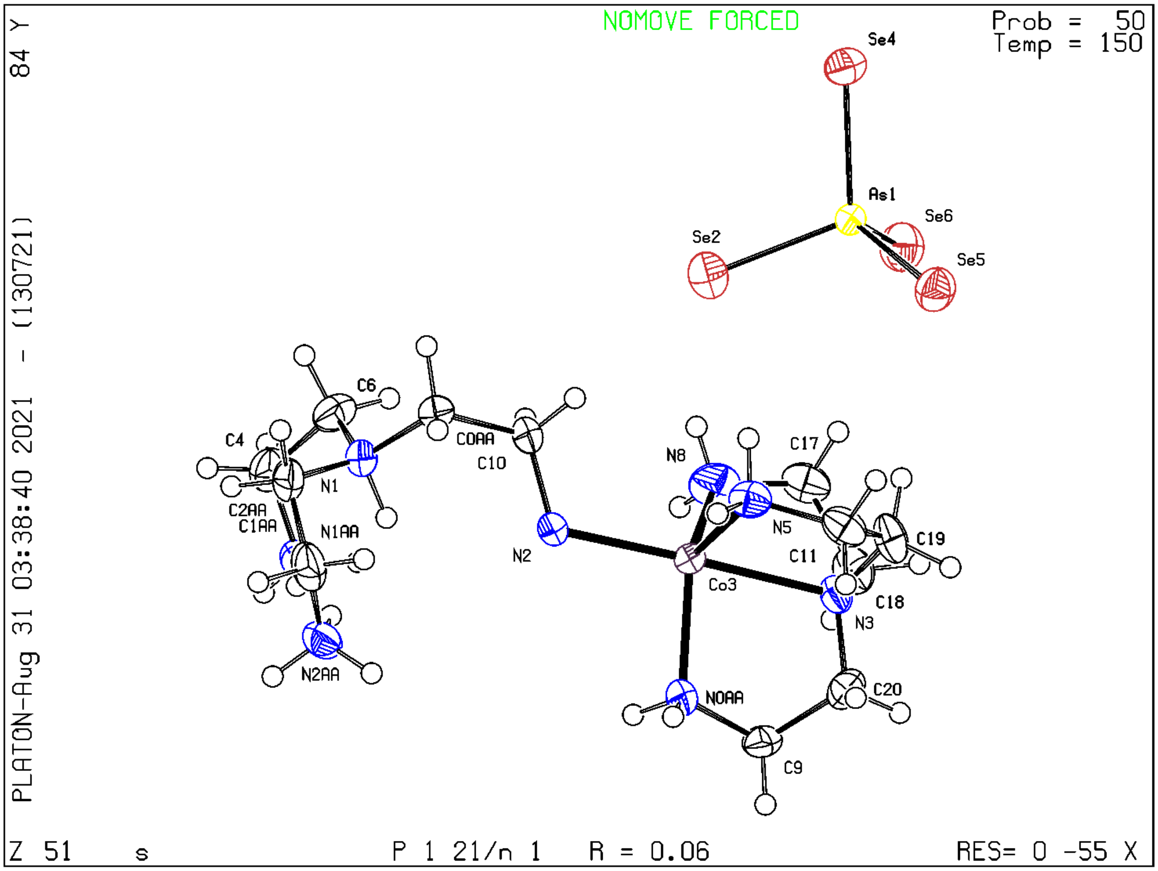

Supplement: Supplementary file 6 [file DataSheet1.PDF]
